# Imaging local diffusion in microstructures using NV-based pulsed field gradient NMR

**DOI:** 10.1126/sciadv.adh3484

**Published:** 2023-08-18

**Authors:** Fleming Bruckmaier, Robin D. Allert, Nick R. Neuling, Philipp Amrein, Sebastian Littin, Karl D. Briegel, Philip Schätzle, Peter Knittel, Maxim Zaitsev, Dominik B. Bucher

**Affiliations:** ^1^ Department of Chemistry, TUM School of Natural Sciences, Technical University of Munich, 85748 Garching, Germany.; ^2^ Division of Medical Physics, Department of Diagnostic and Interventional Radiology, Faculty of Medicine, University of Freiburg, Freiburg, Germany.; ^3^ Department of Sustainable Systems Engineering (INATECH), University of Freiburg, Emmy-Noether-Str. 2, 79110 Freiburg, Germany.; ^4^ Fraunhofer Institute for Applied Solid State Physics, Tullastr. 72, 79108 Freiburg, Germany.; ^5^ Munich Center for Quantum Science and Technology (MCQST), Schellingstr. 4, 80799 München, Germany.

## Abstract

Understanding diffusion in microstructures plays a crucial role in many scientific fields, including neuroscience, medicine, or energy research. While magnetic resonance (MR) methods are the gold standard for diffusion measurements, spatial encoding in MR imaging has limitations. Here, we introduce nitrogen-vacancy (NV) center–based nuclear MR (NMR) spectroscopy as a powerful tool to probe diffusion within microscopic sample volumes. We have developed an experimental scheme that combines pulsed gradient spin echo (PGSE) with optically detected NV-NMR spectroscopy, allowing local quantification of molecular diffusion and flow. We demonstrate correlated optical imaging with spatially resolved PGSE NV-NMR experiments probing anisotropic water diffusion within an individual model microstructure. Our optically detected PGSE NV-NMR technique opens up prospects for extending the current capabilities of investigating diffusion processes with the future potential of probing single cells, tissue microstructures, or ion mobility in thin film materials for battery applications.

## Teaser

Spin defects in diamond detect molecular diffusion through optical magnetic resonance microscopy.

## INTRODUCTION

Molecular and ion diffusion play a major role in many aspects of physics, chemistry, and biology, ranging from nutrient transport in organisms (*1*, *2*), pattern formation (*3*), to the reactivity in chemical reactions (*4*) or the functioning of modern batteries (*5*). Nuclear magnetic resonance (NMR) spectroscopy is one of the prevalent methods for probing diffusion (*6*, *7*), which was described in 1965 by Stejskal and Tanner (*8*). Since then, the technique has developed rapidly and is now used on a daily basis in the form of diffusion-weighted magnetic resonance imaging (MRI) in medicine (*9*–*14*). However, magnetic resonance (MR) methods are limited by the low net nuclear magnetization of the sample, which often leads to the signal-to-noise ratio (SNR) constraining the sample volume of this otherwise powerful technology. Moreover, the spatial resolution in liquid-state MRI techniques is limited by the molecular diffusion that reduces the localization imposed by the applied magnetic field gradient encoding (*15*). In addition, the intrinsic diffusion weighting, which is applied to the sample by the imaging gradients and spoiling gradient pulses themselves, may present challenges in some studies (*16*). For the abovementioned reasons, assessing diffusion with micrometer resolution within thin-film materials, biological tissue, or even for single cells remains extremely challenging for the state-of-the-art NMR methodology.

An elegant solution to overcome these problems is the nitrogen-vacancy (NV) center in diamond which is an atom-sized quantum sensor for magnetic fields (*17*, *18*) (for further details, refer to sections S1 and S2). Because of its spin state-dependent fluorescence, optically detected MR (ODMR) experiments can be performed spatially resolved that translate the local magnetic field into an optical signal. NV centers have been used to conduct NMR experiments on unprecedented length scales (*19*–*23*) and allow the detection of high spectral resolution NMR signals from microscopic sample volumes (*24*–*29*).

**Fig. 1.**
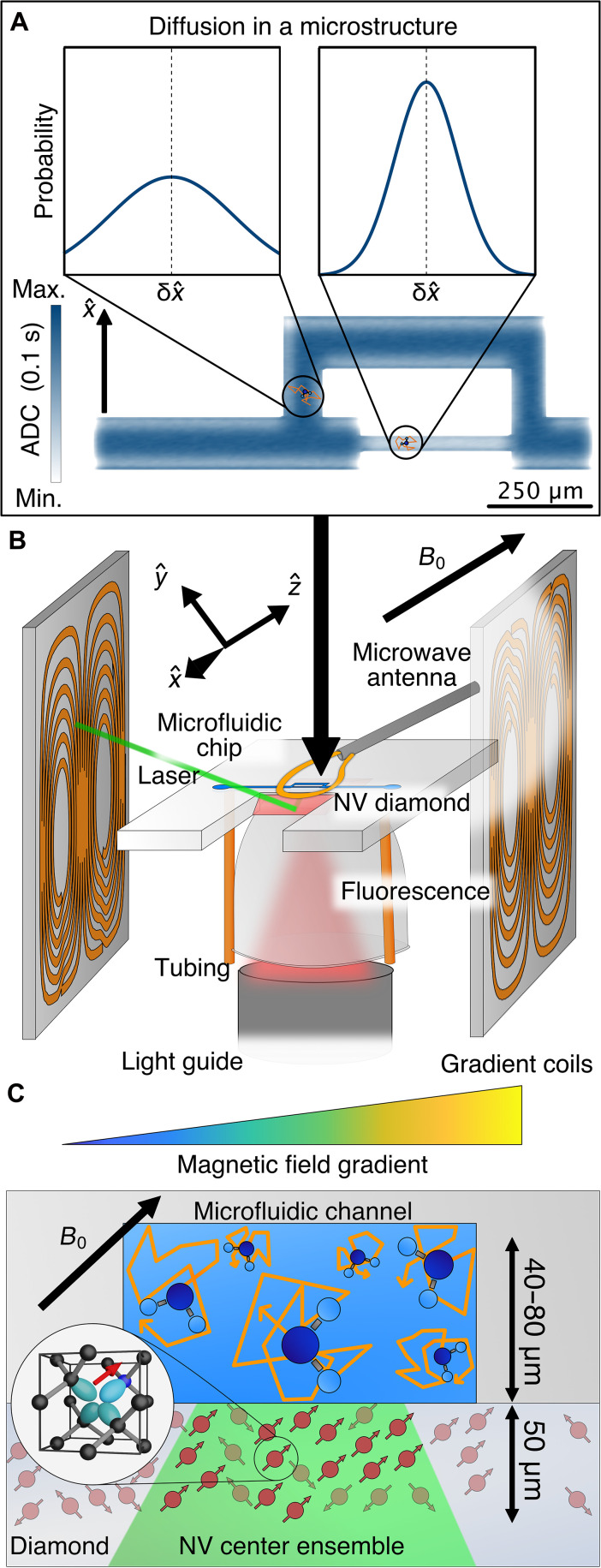
Principles of NV-based diffusion imaging within microstructures. (**A**) Conceptual schematic of diffusion within a microstructure. The apparent diffusion coefficient (ADC) at the two marked locations differs strongly in the $\hat{x}$ direction, because the free diffusion length is on the same scale as the microstructure itself. The probability to find a diffusing particle at a distance $\delta \hat{x}$ from its original position (dashed line) after diffusing for 0.1 s is displayed in the two plots on the top. The microstructure itself is color-coded according to the simulated ADC. (**B**) Experimental setup. A diamond chip (red) with a highly dense surface-doped NV layer is glued into the microfluidic chip (light gray) and placed between three pairs of magnetic field gradient coils. Each pair produces a ${\hat{B}}_{0}$ gradient along one of the cardinal directions $\hat{x}$, $\hat{y}$, and $\hat{z}$. The whole experiment is imaged using an optical microscope from above. A (green) laser enters the diamond chip and excites the NV centers in the surface layer, defining the measurement location [see also (C)]. The red NV fluorescence for signal readout is collected and directed to a photodiode using a liquid light guide. The NV electronic spin, used for the quantum sensing protocol, is driven by a microwave (MW) antenna on top of the microfluidic chip. (**C**) The water sample is confined by a microfluidic channel, whose bottom wall is formed by the NV sensor. Water molecules interacting with the channel walls are hindered in their diffusion and will have a lower ADC. External magnetic field gradients encode the position of the water molecules and allow for the measurement of their ADCs.

This technology is well suited for the investigation of diffusion phenomena on the microscopic level, due to its optical readout, high spatial resolution, and capability of measuring coherent NMR signals. As a rule of thumb, the detection volume of the NV sensor corresponds to the laser spot size and the thickness of the NV layer [details on spatial contributions to the signal in NV-NMR can be found in section S4 and in Bruckmaier *et al*. (*30*)]. In contrast to macroscopic diffusion-based MRI experiments, the NV sensor enables the local detection of the NMR signals on a length scale similar, or smaller than the average distance that a water molecule will have diffused within the timescale of a typical NMR experiment. If the molecule encounters a barrier, then the average displacement is reduced compared to the case of free diffusion. NV-NMR spectroscopy is a promising tool for probing diffusion within microstructures due to its superb localization and potentially higher sensitivity for microscale sample volumes, as shown in Fig. 1A.

In this work, we realize microscopic imaging of molecular diffusion with NV-NMR spectroscopy. We first developed magnetic field gradient coils and designed pulse sequences that combine pulsed field gradients with the NV-NMR detection scheme. This allows us to perform pulsed gradient spin echo (PGSE) experiments to detect diffusion within picoliter sample volumes. In the first series of experiments, we measure water flow within a microfluidic channel. In the second step, a water-soluble polymer is added to probe its influence on water diffusivity. Last, we demonstrate the capabilities of our technique for detecting local water diffusion within a microstructure. Spatially resolved diffusion NV-NMR measurements within a microfluidic model structure show anisotropic diffusion according to the restrictions given by the local geometry and structure.

## RESULTS

### Experimental setup

The experimental setup developed for this publication is depicted in Fig. 1B, which can be split into two parts: the diffusion encoding using magnetic field gradient pulses during a spin-echo sequence and the detection of the corresponding NMR signal with an NV ensemble. We use a highly doped NV layer with a thickness of ~50 μm that allows us to detect NMR signals on a similar length scale that also corresponds approximately to the typical diffusion displacement in our PGSE experiment (*24*, *30*). As a model system, we use microfluidic chips (*29*), where the NV center layer forms the bottom wall of the microfluidic channel. The microfluidic chip is coupled to a syringe pump, allowing for precise control of the sample liquid. For the initialization and readout of the NV center's quantum state for the NMR detection, light from a 532-nm laser is coupled into the trapezoidal diamond via a total internal reflection geometry (*24*). This reduces laser-induced sample damage and heating while increasing the laser intensity at the NV layer (*29*). A compound parabolic concentrator is glued to the bottom side of the NV diamond chip (*31*). It efficiently collects the NV fluorescence that is then directed to a photodiode via a liquid light guide (*32*). The NV diamond and microfluidic structure is imaged from the top, enabling us to correlate an optical image with the PGSE NV-NMR signal, defined by the location of the optical excitation. The free induction decay (FID) of the sample is induced by a radio frequency (RF) pulse, and the corresponding NMR signal is detected via the NV ensemble, which is driven by microwave (MW) pulse sequences. The entire experiment is mounted within a large bore superconducting magnet, which provides a highly homogeneous and stable magnetic field (*B*_0_ ≈ 0.175 T), crucial for the detection of the NMR signal.

**Fig. 2.**
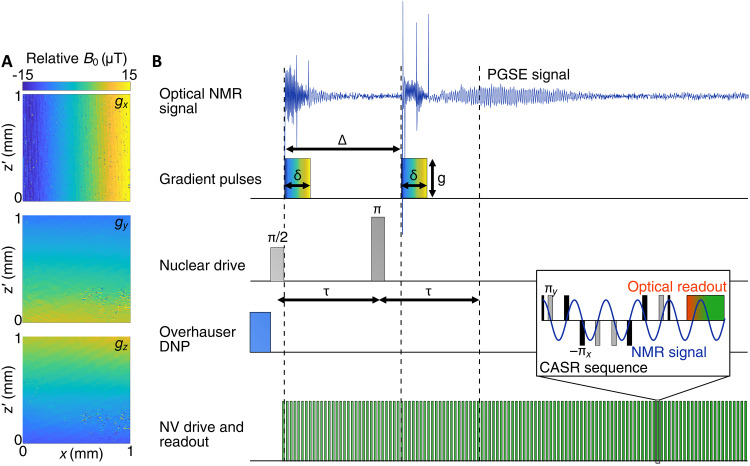
Principle of the PGSE NV-NMR sequence. (**A**) Magnetic field gradients: Measured *B*_0_ gradients along the three cardinal directions (see Fig. 1B) using an NV wide-field magnetic imaging setup. The magnetic field gradients are measured along the diamond surface *x* and *z*′ direction (parallel to the diamond surface). (**B**) Measurement pulse sequence: After hyperpolarizing the sample spins using Overhauser DNP, an RF π/2-pulse at the Larmor frequency of the protons initializes the free induction decay (FID). After a time τ, a π-pulse refocuses the sample nuclear spin magnetization, leading to a spin echo (experimental data in blue). For the PGSE experiment, magnetic field gradient pulses with equal duration δ and strength *g* are applied before and after the nuclear spin π-pulse, separated by a total time Δ. These magnetic field gradient pulses encode the position of the nuclear spins, and any translation or diffusion during the time Δ will reduce the spin-echo signal amplitude. The ADC can be obtained by measuring the spin-echo amplitude as a function of the applied magnetic field gradient strength δ*g*. The spin-echo NMR signal is read out by an NV ensemble using the coherently averaged synchronized readout (CASR) pulse sequence, which consists of a series of dynamical decoupling sequence blocks. Inset: A single dynamical decoupling subsequence, which consists of a train of π-pulses on the NV electronic spin, synchronized to the Larmor frequency of the nuclear spins. Typical parameter values used in this work are δ ≈ 10 ms, Δ ≈ 80 ms and τ ≈ 75 ms, whereas the gradient strength is swept from *g* = 0 to ∼100 μT/mm.

For the PGSE experiment, a set of three pairs of gradient coils ($\hat{x}$, $\hat{y}$, and $\hat{z}$) were designed and fabricated using the openly available gradient coil design tool CoilGen (*33*). These coils have to satisfy unique conditions of NV-NMR spectroscopy, such as the optical access from multiple sides and a gradient along the *B*_0_ field orientation, tilted at an angle of ∼54.7° to the diamond surface normal. This angle is defined by the orientation of the NV centers within our diamond chip and, ultimately, the crystal orientation of the diamond sensor (*30*). For our quantum sensing applications, the external magnetic field *B*_0_ is aligned along this NV axis, negating spin-state mixing, which would otherwise strongly alter the NV center spin dynamics (*34*). The method for finding optimal current carrying surfaces for this setting is described by Amrein *et al.* (*35*). The characterization was performed using ODMR of the NV centers in a wide-field approach (*36*), extracting the relative ${\hat{B}}_{0}$ amplitudes over the diamond by measuring the NV center Zeeman splitting, resulting in experimentally assessed gradient sensitivities of $g_{x}\approx 29.74\pm 0.09\;\frac{\mu T}{A\;\mathrm{mm}}$, $g_{y}\approx 25.92\pm 0.09\;\frac{\mu T}{A\;\mathrm{mm}}$, and $g_{z}\approx 23.27\pm 0.06\;\frac{\mu T}{A\;\mathrm{mm}}$, respectively (Fig. 2A). In combination with the available current sources and under the constraints of air cooling in our proof-of-concept experiments, we were able to reach gradient strengths of ~$100\frac{mT}{m}$, which may appear rather weak to the standards of NMR microscopy but is on par with top-performance whole-body clinical MRI scanners.

### Pulse sequence and theory

For the NMR signal detection, we use the coherently averaged synchronized readout (CASR) method (see Fig. 2B) (*24*). It consists of a train of dynamical decoupling sequences, that are synchronized to the sample's FID using an external clock. The detected signal of the optical NV readouts using CASR is an aliased version of the NMR signal. A more in-depth explanation of the sensing scheme is described in section S2 and in Glenn *et al.* (*24*). All experiments described in this work were conducted on proton spins in water, which were detected at an NMR resonance frequency of ∼7.45 MHz (*B*_0_ ≈ 0.175 T). To increase the NMR signal and reduce the averaging time, Overhauser dynamic nuclear hyperpolarization (ODNP) was used in all experiments by adding stable radicals to our water sample and irradiating with microwaves before each NV-NMR measurement (*27*).

As noted above, the diffusion NMR method used here is called pulsed gradient spin echo (PGSE) (*8*). This sequence is a modification of the classic spin-echo experiment, where before and after the refocusing π-pulse, two identical spatially varying *B*_0_ gradient pulses are applied. The magnetic field gradient causes a spatially dependent Larmor frequency, which encodes the position of the nuclear sample spins. The first gradient pulse leads to a relative phase accumulation of each individual sample spin depending on its position, and the second gradient pulse leads to an inverse phase accumulation or refocusing up to the amount each spin has diffused along the gradient in the time between the two pulses. In the limit where the pulsed gradient amplitude is much larger than the constant background gradient of the magnetic field, the apparent diffusion coefficient (ADC) can be extracted by sweeping the strength of the applied gradient according to

$$\mathrm{ADC}=-\ln(A/A_{0})\;{\left[{\left(\delta \;g\;\gamma \right)}^{2}\;\left(\text{Δ}-\frac{\delta }{3}\right)\right]}^{-1}$$ (1)

where *A* and *A*_0_ are the spin-echo amplitudes with and without gradient pulses, respectively; δ is the duration; Δ is the spacing of the gradient pulses; *g* is the strength of the applied gradient pulses; and γ is the gyromagnetic ratio of the sample spins (*8*). Figure 2B and section S10 show the corresponding pulse sequence and an experimental dataset. The PGSE measurement is then repeated multiple times for averaging, and a linear fit is performed to the logarithmic scale of the resulting signal amplitudes to extract the ADC. The details are described in Material and Methods and further in Kingsley (*37*). For restricted diffusion, as it is the case in our microfluidic channel, a slightly modified model including tensor properties of diffusion needs to be used to determine the ADC, which can be found in section S4. Individual tensor elements can be measured, by changing the direction of the first and/or second gradient pulse. The gradient directions used in this work are (i) parallel to *B*_0_ ($\hat{z}$), (ii) orthogonal to *B*_0_ and parallel to the diamond surface ($\hat{x}$), and (iii) the remaining direction at a ~35.26° angle to the diamond surface normal ($\hat{y}$), as depicted in Fig. 1B.

### Velocimetry measurements

**Fig. 3.**
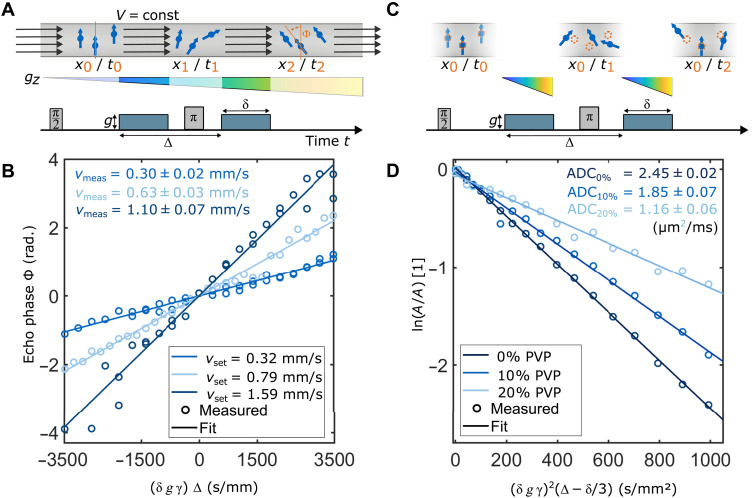
Measuring water flow and diffusion using PGSE NV-NMR. (**A**) Schematic of sample spins moving in a microfluidic channel during a velocimetry measurement. During the PGSE experiment, a constant and laminar flow is applied, which leads to an equal translation of all sample molecules from their initial, *t* = *t*_0_, location *x*_0_ to their final position *x*_2_ at *t* = *t*_2_. This leads to an equal phase shift ϕ for each spin within the sample, which depends on the translated distance between the two gradient pulses and the strength (*g*) and duration (δ) of the individual pulses. The PGSE sequence is sketched in the bottom, and the absolute gradient strength of each pulse is color-coded. (**B**) Experimental data (circles) and fits (lines) for three different flow rates. Increasing the gradient amplitude leads to a larger phase accumulation due to the flow in the channel. (**C**) Molecular diffusion leads to a random displacement of the sample spins, which effectively attenuates the amplitude of the PGSE signal. The PGSE sequence is sketched in the bottom of the figure. (**D**) Three PGSE measurements (sweeping the gradient strength) of different concentrations of polyvinylpyrrolidone (PVP) K90 in water. The normalized spin-echo amplitudes are displayed as circles and linear fits as dashed lines.

In the first set of experiments, we used our PGSE NV-NMR setup to measure the flow velocity of water within a microfluidic channel. Assuming a homogeneous flow profile, each water molecule will have moved the same distance along the gradient during time Δ. This causes a common relative phase shift ϕ (Fig. 3A) of the nuclear spins, which can be detected with NV-NMR spectroscopy (*24*). Including laminar flow into Eq. 1, the combined effects of diffusion and translation on the sample magnetization can be described as (*8*, *38*)

$$A/A_{0}=\exp[iv\gamma g\delta \text{Δ}-D\;{\left(\gamma g\delta \right)}^{2}(\text{Δ}-\delta /3)]$$ (2)

where *v* is the flow velocity within the channel and *i* is the imaginary unit. The signal phase ϕ = *v*γ*g*δΔ can be extracted from the experimental data via the imaginary and real part of the spin echo’s Fourier transformation. We note that conventional NMR methods exist, which directly measure the full propagator described in Eq. 2 (*15*). Plotting the phase ϕ against the magnetic field gradient strength allows us to determine the velocity from a linear fit (*39*). Because of a complex interplay between the quadratic flow profile in our microfluidic channel and the inhomogeneous spatial sensitivity of NV-NMR spectroscopy (*30*), the recorded phase shift is not a simple linear relation to the set mean phase shift of the sample. The nonlinearity was corrected by using numerical simulation, as described in section S5.

The experiments were conducted in a straight microfluidic channel with dimensions of 80 μm (orthogonal to the diamond surface) by 100 μm (along the $\hat{x}$ direction) by 2000 μm (along the diamond surface) (*29*). The experimentally measured flow rates by PGSE NV-NMR were 0.30 ± 0.02 mm/s, 0.63 ± 0.03 mm/s, and 1.10 ± 0.07 mm/s, which are slightly but consistently lower than the parameters set at the syringe pump (Table 1 and Fig. 3B). This can be explained by an additional layer of glue in between the diamond and the microfluidic chip, increasing the effective volume of the channel: The flow rate in the microfluidic channel is calculated from the flow rate set at our pump, given in units of $\frac{\mathrm{volume}}{\mathrm{time}}$. This is divided by the intended cross section of the microfluidic channel, resulting in the flow rates *v*_set_. Any difference between the cross section of the microfluidic channel in the experiment and as designed would lead to a proportional offset between measured and set flow rates.

**Table 1.** Results of the PVP diffusion measurements. Literature (*40*–*42*), simulated, and experimental values of the ADC for three different concentrations of PVP in water (w/w) with their respective uncertainties.

ADC*_z_* (μm^2^/ms) at ∼25°C	0% PVP	10% PVP	20% PVP
Literature	2.31	1.81	1.37
Simulation	2.14	1.69	1.13
Experimental result	2.45 ± 0.02	1.85 ± 0.07	1.16 ± 0.06

### Diffusion measurements

In the second set of experiments, we measured the diffusion coefficient of water in the microfluidic channel. In contrast to the laminar flow in the previous experiment, diffusion leads to a random motion and a reduction of the spin-echo amplitude as a function of gradient strength (Fig. 3C). We used water with varying concentrations of an organic polymer polyvinylpyrrolidone (PVP) K90 (0, 10, and 20% w/w) at *T *≈ 25°C, to modify the diffusivity of water, similar to previous reports (*40*–*43*). Because we measure the water diffusion within a microfluidic channel, the free diffusion will be attenuated by its boundaries. For that reason, we chose to sweep the amplitude of the **gradient *g*_z_ to measure the ADC, because the diffusion along this direction is the least restricted and, therefore, the closest to the values reported in the literature. Nevertheless, the boundaries of the microfluidic channel will reduce the ADC’s diagonal elements, compared to the free diffusion case. Therefore, we simulated the expected ADC on the basis of the literature values as described in section S4. The resulting data can be found in Table 1 and in Fig. 3D. The expected and simulated values are in good agreement with the values obtained from PGSE NV-NMR measurements. The remaining discrepancy between measured and simulated parameters can be explained by possible sample heating, as discussed in section S7, which affects the diffusion in solutions with higher concentrations of PVP to a lesser degree (*43*).

### Investigating the time dependence of the ADC

**Fig. 4.**
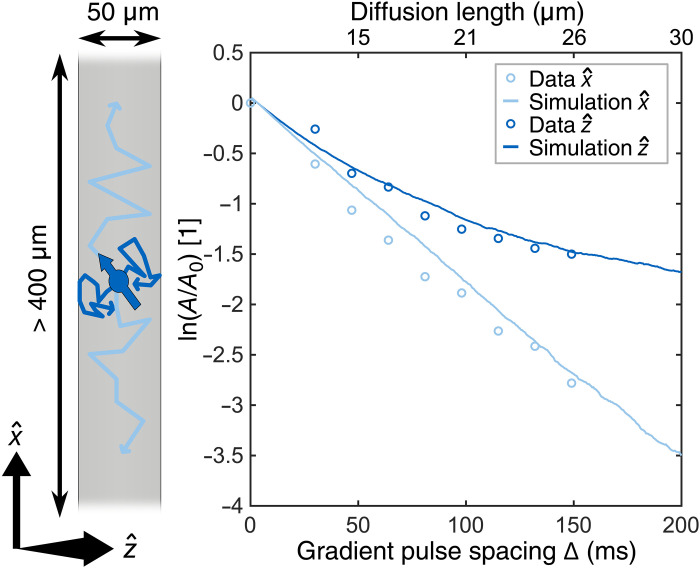
Investigating the time dependence of the ADC. Left: A sketch of diffusing sample molecules in the microfluidic channel. The diffusion along the $\hat{z}$ direction is constrained, leading to an increased number of molecules interacting with the channel boundaries as the gradient pulse spacing Δ is increased. In contrast, there are no effective restrictions in the $\hat{x}$ direction. Right: Simulation (line) and experimental (circles) data of a PGSE amplitude is plotted on the *y* axis. For reference, the expected root mean square distance diffused during Δ for the case of free diffusion is given on the top of the plot. Along the $\hat{x}$ direction (light blue), both simulation and experimental data predict a linear decay of the spin-echo amplitude in the logarithmic scale, as is the case for free diffusion; see Eq. 1. Along the $\hat{z}$ direction (dark blue), the diffusion is restricted by the channel walls, and an increasing number of molecules interact with the channel walls, and the ADC decreases over time. This leads to a slower decay of the spin-echo amplitude.

**Fig. 5.**
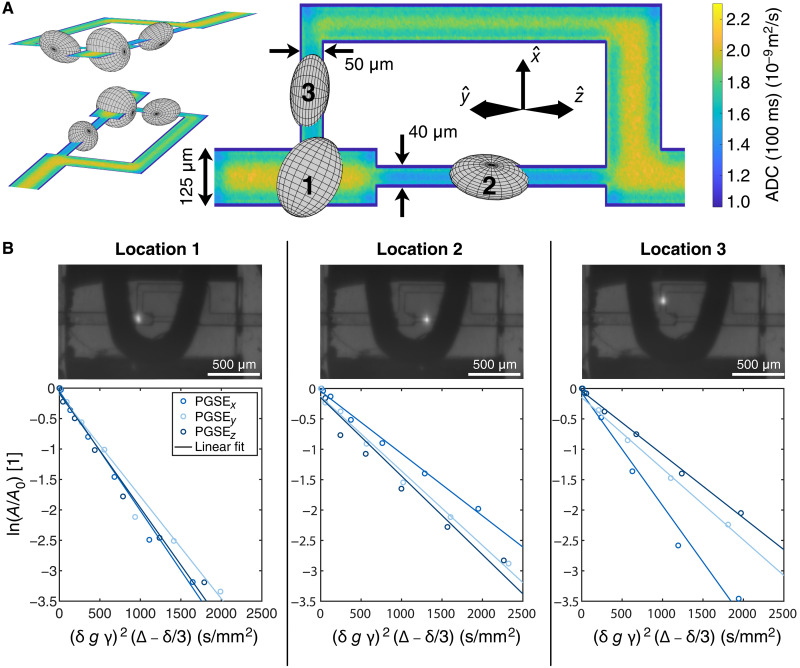
Spatially resolved PGSE NV-NMR experiments within microfluidic structures. (**A**) Visualization of the measured diffusion tensors in our microfluidic channel. The size of the ellipsoid corresponds to the strength of the ADC in this direction. Three different perspectives of the ellipsoids are displayed for a better visualization. The color of the microfluidic channel corresponds to the trace of the numerically simulated diffusion tensor after Δ = 100 ms. (**B**) Photographs of the investigated locations (top) taken with a camera. Exemple datasets for each of the three locations (bottom); the diffusion is close to free diffusion for all directions at location 1, while, at locations 2 and 3, the $\hat{x}$ and $\hat{y}$ or $\hat{z}$ directions are more restricted, respectively.

Having established the ability to perform PGSE experiments in combination with NV-NMR spectroscopy, we investigate the effects of the restricted diffusion in one of our microfluidic channels. The one-dimensional ADC can be defined as

$$\mathrm{ADC}=\frac{\left\langle x^{2}\right\rangle }{2\text{Δ}}$$ (3)

where Δ is the free diffusion time during our PGSE experiment. In the case of free diffusion, this relationship is constant, and the ADC is independent of the time Δ. In the case of restricted diffusion, the mean square distance diffused ⟨*x*^2^⟩ is limited by the length scale of the restriction. As the free diffusion time Δ increases, more and more molecules will interact with the confinement boundaries, and the ADC will tend to 0. To investigate this phenomenon, we performed experiments in a microfluidic channel with strong confinement along the direction perpendicular to the channel and effectively no confinements along the longitudinal direction. The experimental results were verified by numerical simulations; both data are shown in Fig. 4. An image of the corresponding microfluidic channel can be found in Fig. 5 (location 3). Along the longitudinal direction, we expect to find unrestricted diffusion of the sample molecules, leading to linear dependence of the spin-echo amplitude on the free diffusion time in the logarithmic scale (Eq. 1). This is verified by both experimental data and the numerical simulations (Fig. 4). Along the perpendicular direction, we expect a slower signal decay, because the root mean square distance diffused along this direction is limited, and, as described above, the ADC will decrease with increasing Δ. This is clearly evident in both the experimental data and the conducted simulations. The simulations are described in detail in Materials and Methods and in section S4.

### Measuring spatially resolved anisotropic diffusion

Having demonstrated the effect that the microfluidic channel walls have on the free diffusion of the sample molecules, we continue to probe water diffusion spatially resolved within microstructures. For that purpose, we designed and fabricated a microfluidic structure with different channel sizes and orientations. Because of the optical readout of the PGSE NV-NMR signal, any location within this structure can be probed by moving the NV excitation laser (Fig. 5, A and B). Our current setup has a field of view of approximately 1 mm^2^ and hence requires scanning with the laser location for spatially resolved measurements. Future developments may use wide-field imaging to alleviate this issue (*36*).

For an estimation of the expected ADC within our microfluidic structure, we simulated the ADC for each point using particles undergoing a random walk. Because of the small length scales, the ADC along the different cardinal directions can vary significantly (Fig. 5). Then, we performed PGSE NV-NMR experiments at three different locations and along six different directions within this structure. From these six noncolinear measurements, the diffusion tensor is calculated according to Kingsley (*37*). The resulting tensors are depicted as ellipsoids in Fig. 5A. The ADC changes depending on the channel dimensions at the location of the laser spot within the structure, in accordance with our simulation (Fig. 5B). The difference is most pronounced when measurement locations 2 and 3 are compared. In location 2, the microfluidic channel has a cross section of 40 μm by 40 μm, meaning the diffusion is strongly constrained in the $\hat{x}$ direction, while it is close to free along the $\hat{z}$ direction. On the other hand, at location 3, the channel has a cross section of 50 μm by 80 μm. Thus, diffusion along the $\hat{x}$ direction can be considered free, and the shortest direction perpendicular to $\hat{x}$ is constrained (~50 μm). We expect the eigenvectors of the ADC tensor to be orthogonal to the walls of the approximately cuboid microfluidic channel. The tensors calculated from our measurements (Fig. 5) are close to this expected orientation (section S8). The resulting ADCs are in good agreement with the simulation (Table 2).

**Table 2.** Results of the spatially resolved PGSE NV-NMR experiments within the microfluidic structure. Measured and simulated elements of the measured ADC tensor for each of the locations investigated. An exemplary dataset and the full tensor including the off-diagonal elements can be found in section S8.

ADC [μm^2^/ms] at ∼25°C		Location 1	Location 2	Location 3
ADC*_x_*	Simulated	2.19 ± 0.10	1.06 ± 0.10	2.29 ± 0.10
	Measured	2.24 ± 0.13	1.19 ± 0.13	2.24 ± 0.07
ADC*_y_*	Simulated	1.92 ± 0.10	1.75 ± 0.10	1.36 ± 0.10
	Measured	1.93 ± 0.16	1.67 ± 0.32	1.39 ± 0.29
ADC*_z_*	Simulated	2.01 ± 0.10	2.03 ± 0.10	1.16 ± 0.10
	Measured	2.16 ± 0.15	2.10 ± 0.31	1.18 ± 0.05

## DISCUSSION

In this work, we have demonstrated spatially resolved PGSE NMR experiments within microstructures using NV centers in diamond. We note that the spatial resolution has not yet reached any physical limitations. Higher spatial resolution can be achieved by decreasing the thickness of the diamond’s NV center–doped layer and reducing the diameter of the excitation laser beam. In our current experimental setup, both the NV center–doped layer and the diameter of the laser location are on the order of ~50 μm, limiting our spatial resolution to the same order of magnitude (*30*). Reaching the optical diffraction limit is feasible, although with the drawback of highly reduced sensitivity, that would lead to longer averaging times, as discussed in section S6. Thus, this technique could enable quantifying the diffusion properties of basic microstructural building blocks on the single cell level, which could help to validate current models in medical MRI (*9*, *14*). However, for biological applications, ODNP, as used in our study, is not recommended, because of the need for polarizing agents within the sample and strong MW fields. Alternatives are increasing the magnetic field strength* B*_0_ (e.g., to 1 T) or using other biocompatible hyperpolarization methods (*44*) [for instance, dissolution DNP or parahydrogen-induced hyperpolarization (*25*)]. The latter methods allow for high signal enhancements by relying on promptly injected hyperpolarized samples and, in combination with NV-NMR, could potentially allow for probing the diffusion of metabolites in single cells.

Another unique feature of our method is the possibility of applying very strong magnetic field gradients (*45*, *46*). Because of the small length scale of NV-NMR, gradient coils can be miniaturized and thereby can achieve up to 10 mT/μm (*47*, *48*). For such extremely high gradient fields, concomitant field components that occur as an unavoidable consequence of Maxwell’s equations may require corrective actions, e.g., by designing specialized compensated pulse sequences (*49*) or by increasing the main magnetic field strength *B*_0_. These technical developments may provide unique insights, e.g., in detecting slowly diffusing spins (*50*, *51*) (such as Li ions in solid state materials), in detecting liquid or moleclar diffusion on very small length scales, or elucidating the origin of “dot-compartment,” small diffusion–restricted spaces in tissues, which are currently discussed in literature (*52*).

In summary, we have developed a powerful NV-based NMR method, which enables us to image diffusion on microscales. The technique allows for the local detection of water flow and diffusion within microscopic sample volumes. Last, we demonstrated the capability to measure the ADC spatially resolved within a model microstructure in three directions, which showed restriction in diffusion due to the local geometry. Although our current spatial resolution is comparable with published conventional diffusion MRI results (*10*, *53*, *54*), we are limited by the optical resolution rather than the spatial encoding with magnetic field gradients (*15*). We anticipate that further technical improvements will allow us to approach a spatial resolution of about one micrometer. This advancement will shed light on microstructure diffusion by decoupling the long-standing link between spatial encoding and diffusion weighting in MR. Our technique and experiments mark a major milestone toward probing single cells, tissue microstructures, and ion-conducting materials in energy research.

## MATERIALS AND METHODS

### Experimental setup

Schematics of the experimental setup with its optics and electronics is shown in section S3 in figs. S3 to S6. An electronic grade, single-crystal, 100-oriented diamond (2 mm by 2 mm by 0.5 mm; Element Six, Oxford, UK) was overgrown with a ∼19–parts per million (ppm) nitrogen-doped ^12^C and ^15^N isotopically enriched diamond layer with a thickness of ∼50 μm by the Fraunhofer Institute for Applied Solid State Physics (Freiburg, Germany) as described by Schätzle *et al.* (*55*). This particular thickness of the nitrogen-doped layer was chosen for an optimized SNR obtained by simulations of our microfluidic channels (*29*). The diamond was then electron irradiated (1.5 × 10^18^/cm^2^, 1 MeV) and annealed to increase the nitrogen to NV-conversion efficiency (6 hours at 400°C, 6 hours at 800°C, and 2 hours at 1200°C, at a pressure of <10^−^^7^ mbar) and cut into a trapeziodal shape. Ramsey and Hahn echo experiments were used to measure an NV-ensemble $T_{2}^{*}$ dephasing time of ∼0.65 μs and *T*_2_ decoherence time of ∼9 μs, respectively. The diamond chip was glued into a microfluidic chip designed in-house and fabricated by LightFab GmbH (Aachen, Germany) using Norland Optical Adhesive 68 ultraviolet-curing glue (*29*). The assembled microfluidic chip with diamond was positioned in a custom-built superconducting magnet (3T-215-RT, Superconducting Systems Inc., Billerica, USA), and one of the four possible NV orientations with the diamond lattice was aligned with the external magnetic field (*B*_0_ ≈ 0.175 T). Flow pumps (AL-1000, World Precision Instruments, Sarasota, USA) were used to control the flow velocity within the microfluidic channel. The NV diamond’s fluorescent light was collected using a compound parabolic concentrator glued to the bottom of the microfluidic chip (designed in-house and fabricated by Süd-Optik Schirmer GmbH, Kaufbeuren, Germany). The output was attached to a custom-made liquid light guide (Lumatec GmbH, Munich, Germany), which directs the fluorescence through a long pass filter (BLP01-647R-25, Edge Basic 647 Long Wave Pass, Semrock, Rochester, USA) onto a balanced photodiode (PDB210A, Thorlabs, Bergkirchen, Germany). A reference laser beam was used for efficient laser noise cancellation. The photodiode’s voltage was read out using a data acquisition unit (NI USB-6281, National Instruments, Austin, USA).

The NV center spins were initialized using a 532-nm laser (Laser Quantum Opus 53, Novanta Photonics, Wackersdorf, Germany) with a power of about ∼380 mW. Initially, the laser passes an opto-acoustic modulator (3260-220, Gooch and Housego, Ilminster, UK) to generate pulses of a typical length of 5 μs. A multiorder half-wave plate (WPMH05M-532, Thorlabs, Bergkirchen, Germany) was used to adjust the polarization of the laser light for efficient NV excitation. Last, the laser beam was expanded (BE02-05-A, Thorlabs, Bergkirchen, Germany) and focused onto the diamond using a *f* = 250-mm lens (LA1433-B-ML, Thorlabs, Bergkirchen, Germany), resulting in a beam diameter of 1/*e*^2^ ≈ 45 μm. The position of the laser location within the microstructure was imaged from the top by a camera (a2A3840-45umBAS, Basler, Ahrensburg, Germany).

The whole experimental sequence was controlled by an arbitrary waveform generator (AWG70002B, Tektronix, Beaverton, USA). It synchronizes all other devices (signal sources, switches, data acquisition unit, and opto-acoustic modulator) via synchronized transistor-transistor logic signals (fig. S4 in section S3). The NV pulse sequence is programmed and uploaded with a 500-MHz carrier frequency and upconverted using an IQ mixer (MMIQ0218LXPC, Marki Microwave, Morgan Hill, USA) and an MW signal source (SMB100A, Rhode und Schwarz, Munich, Germany). The resulting MW pulses were then amplified using a broadband 50-W amplifier (AMP1016, Exodus, Las Vegas, USA) and delivered using a home-built MW antenna (*56*). An RF source (LXI DG1022, Rigol, Suzhou, China) was amplified (LZY-22+, Mini-Circuits, Brooklyn, USA) and connected to two coils in a Helmholtz geometry with radius *R* = 1.5 cm for driving the sample nuclear spins with Rabi frequencies up to ~6.3 kHz. An additional coil for calibration purposes was connected to another RF source (LXI DG1022, Rigol, Suzhou, China), to determine the sensitivity of our experiment as described by Glenn *et al.* (*24*). A third RF source (LXI DG1022, Rigol, Suzhou, China) was used to generate the gradient pulses that were fed into a bipolar power supply (BOP 5-20DL, Kepco, Naju, South Korea) capable of ±20 V and ±5 A, which, in turn, was connected to the gradient coils (Beta-Layout, Aarbergen, Germany). The microfluidic chip, MW, RF, and gradient coils were all mounted on a custom-designed, three-dimensionally printed sample holder (grey v4 resin, Form 3, Formlabs, Somerville, USA). Photos and schematic of the setup and the assembly is depicted in figs. S5 and S6 in section S3. To increase the NMR signal from the water sample, Overhauser DNP was used (*27*). In all experiments, a 10 mM concentration of 4-hydroxy-2,2,6,6-tetramethylpiperidin-1-oxyl (TEMPOL) was added to the respective sample. TEMPOL is a stable radical, which, under continuous, strong, and resonant MW radiation (0.3 s), can hyperpolarize the nuclear sample spins, leading to a ~200-fold increase in the NMR signal strength (*27*).

### Chemicals

The PVP K90 (PVP, 81440, Sigma-Aldrich, St. Louis, USA) and the 4-hydroxy-2,2,6,6-tetramethylpiperidin-1-oxyl (TEMPOL, 581500, Sigma-Aldrich, St. Louis, USA) were purchased from Sigma-Aldrich and used without further purification steps; the chemical structures are depicted in section S9. The solutions were prepared using deionized water with a resistivity of 18.2 megaohms·cm (MilliporeSigma, Burlington, USA).

### Gradient coil design

A MATLAB-based software package (*33*) based on the stream function method (*57*) was used for the design of the gradient system. A biplanar configuration was chosen for the geometry because it allows for better access for the fluorescence optical readout path compared to other geometries. Searching for a suitable biplanar configuration, several geometrical parameters were investigated such as plate size, plate distance, and plate orientation, and, after evaluation, a solution with a plate size of 50 mm, a plate distance of 30 mm, and an atypical azimuth plate tilt of ~35.26° against the *B*_0_ magnetic field was selected for printed circuit board fabrication. Although the value of ~35.26° for the azimuthal inclination is not optimal for the gradient’s strength (the optimum is found at 55°), the gradient plates mounted vertically present a reasonable compromise between the achievable performance and the compatibility with the NV-NMR experimental setup. Because the gradient coils are only added for diffusion weighting, thermal limitations are not expected if the duty cycles of the used NMR sequence are sufficiently low. More information on the design can be found in Amrein *et al.* (*35*).

### Microscale NV-NMR using the CASR pulse sequence

For this work, the universally robust dynamical decoupling sequence (*58*) containing 12 π-pulses were used, with typically 50 repetitions, leading to ≈600 π-pulses per measurement step. A typical duration of each π-pulse was ~30 ns. In the case of the CASR pulse sequence, a detuning δ*f* to the peak frequency *f*_0_ is detected typically in the range of ∣δ*f*∣ < 3000 Hz (*24*). The typical ac sensitivity and volume normalized ac sensitivity of our experiment were ~20 pT/$\sqrt{\mathrm{Hz}}$ and ∼5.6 nT$\sqrt{\mu m^{3}}/(\sqrt{\mathrm{Hz}})$, respectively. An example of a CASR measurement using the universally robust dynamical decoupling sequence is depicted in Fig. 2. More information on the CASR method can be found in Glenn *et al.* (*24*) and section S2.

### PGSE NV-NMR pulse sequence

Typical parameter values used for the PGSE sequence were δ ≈ 10 ms, Δ ≈ 80 ms, τ ≈ 75 ms and gradient strength from *g* ≈ 0 to 100 mT/m. Experiments were typically averaged 100 times each, usually waiting a total of 3 s in between averages, to allow relaxation of the sample nuclear spins to thermal equilibrium. The typical single-shot SNR of a hyperpolarized water NV-NMR signal in our experiments was ~100. Typical coherence and relaxation times of the water proton spins were $T_{2}^{*}$ ≈ 60 ms, *T*_2_ ≈ 80 ms, and *T*_1_ ≈ 300 ms. $T_{2}^{*}$ is likely limited by magnetic field inhomogeneities of our experimental setup, while *T*_2_ and *T*_1_ were limited through the addition of TEMPOL to the solution.

### Wide-field gradient imaging using CW-ODMR

The magnetic field gradients, shown in Fig. 2A, are measured by wide-field dc magnetic imaging using continuous-wave ODMR (CW-ODMR) (*36*). We used an electronic grade diamond chip (1.9 mm by 1.9 mm by 0.5 mm) with a 14-μm-thick ^12^C and ^15^N isotopically enriched, nitrogen-doped layer (nitrogen concentration, ∼2.3 ppm), which was electron-irradiated and annealed to increase the nitrogen to NV center conversion rate. An external magnetic field *B*_0_ of ∼ 4.4 mT is applied along the NV symmetry axis that lifts the degeneracy of the *m*_s_ = ±1 states. For NV-excitation, green laser light (Sapphire LPX, Coherent, Santa Clara, USA) is used to fully illuminate the diamond chip (∼600 mW). NV spin *m*_s_ = 0 → ±1 transitions are probed by sweeping the frequency of an applied MW field in 400 steps (200 steps for each transition). The MW frequencies were produced by a signal source (SMB100A, Rhode und Schwarz, Munich, Germany), amplified (ZHL-16 W-72+, Mini-Circuits, Brooklyn, USA), and delivered to the diamond sample by an homebuilt antenna. The NV fluorescence was passed through a longpass filter (BLP01-647R-25, Edge Basic 647 Long Wave Pass, Semrock, Rochester, USA) and imaged on a camera (a2A1920-160umBAS, Basler, Ahrensburg, Germany) with a magnification of x2.75. For the measurements, 4 × 4 pixels were binned on the camera, resulting in 480 × 304 data points. Each data point was recorded with an exposure time of 600 μs and averaged 800 times. Thus, we acquire an image stack, where each pixel stack corresponds to a single CW-ODMR spectrum. Four different CW-ODMR spectra are recorded with and without (background) applying a current of 1 A to the $\hat{x}$, $\hat{y}$, and $\hat{z}$ gradient coils. The gradient fields along *B*_0_ are obtained by fitting (double Lorentzian functions) the NV resonance lines of the collected data after subtracting of the background field *B*_0_. Magnetic field values were calculated for each pixel stack from the splitting of the *m*_s_ = ±1 states (2γ*B*_0_), resulting in a two-dimensional magnetic field map. The fitted gradients in the *g_y_* and *g_z_* directions were corrected using factors of $\frac{1}{\sin(35.26^{\circ })}$ and $\frac{1}{\cos(35.26^{\circ })}$, respectively, because these gradient directions are not parallel to the diamond surface. Any constant offset produced by the gradient coils can be neglected, because it will have a negligible effect on the echo amplitude of the sample magnetization.

### Data analysis

The PGSE experiments were typically averaged 100 times. The data from the end of the second magnetic field gradient pulse to the point in time where the spin echo signal was below the noise floor, usually after about 250 to 350 ms, were used for analysis. This window was zero filled to a total of three times the initial length and was Fourier-transformed. The NMR signal peak was integrated, and the resulting data were normalized to the data point with the highest signal amplitude, taking into account the possibility of constant background gradients. Last, the whole dataset was fitted with the function *G*

$$G(D,g,\delta g)=\ln(\exp\{-D\;{\left[\delta \gamma (g-\delta g)\right]}^{2}\;[\text{Δ}-\delta /3]\}+\mathrm{Offset})$$ (4)

where Δ, δ, and γ are known and the gradient amplitude *g* is swept. δ*g* is a fit parameter that takes constant magnetic field inhomogeneities caused by magnetic susceptibility mismatches between sample, microfluidic, and diamond chip into account. For the calculation of the ADC tensor, we followed Kingsley (*37*).

### Simulation of diffusion in a restricted volume

The simulations are based on a random walk of individual sample particles, in a defined, microscale volume. A similar technique is used by Cartlidge *et al.* (*59*) to simulate the diffusion induced relaxation in porous media. In each iteration, a Gaussian-distributed, random distance in an equally distributed random direction is chosen, and the particle is moved accordingly. If the path hits a boundary, the particle is reflected inward. At each time step the root mean square distance traveled can be calculated, which is directly related to the ADC. For more information, see section S4 and (*30*). 

## Supplementary Materials

### This PDF file includes:

Sections S1 to S10 Figs. S1 to S15 References

## References

[1] R. Spector, Nutrient transport systems in brain: 40 years of progress. J. Neurochem. 111, 315–320 (2009). 19686385 10.1111/j.1471-4159.2009.06326.x

[2] V. Gulani, A. G. Webb, I. D. Duncan, P. C. Lauterbur, Apparent diffusion tensor measurements in myelin-deficient rat spinal cords. Magn. Res. Med. 45, 191–195 (2001). 10.1002/1522-2594(200102)45:2<191::aid-mrm1025>3.0.co;2-9 11180424

[3] A. N. Landge, B. M. Jordan, X. Diego, P. Müller, Pattern formation mechanisms of self-organizing reaction-diffusion systems. Dev. Biol. 460, 2–11 (2020). 32008805 10.1016/j.ydbio.2019.10.031 PMC7154499

[4] R. M. Noyes, Models relating molecular reactivity and diffusion in liquids. J. Am. Chem. Soc. 78, 5486–5490 (1956).

[5] T.-F. Yi, T.-T. Wei, Y. Li, Y.-B. He, Z.-B. Wang, Efforts on enhancing the li-ion diffusion coefficient and electronic conductivity of titanate-based anode materials for advanced li-ion batteries. Energy Stor. Mater. 26, 165–197 (2020).

[6] P. T. Callaghan, *Translational Dynamics and Magnetic Resonance* (Oxford Univ. Press, 2011).

[7] J. J. Neil, Measurement of water motion (apparent diffusion) in biological systems. Magn. Reson. A. Bridg. 9, 385–401 (1997).

[8] E. O. Stejskal, J. E. Tanner, Spin diffusion measurements: Spin echoes in the presence of a time-dependent field gradient. J. Chem. Phys. 42, 288–292 (1965).

[9] E. Fieremans, H.-H. Lee, Physical and numerical phantoms for the validation of brain microstructural MRI: A cookbook. Neuroimage 182, 39–61 (2018). 29920376 10.1016/j.neuroimage.2018.06.046 PMC6175674

[10] G. A. Johnson, Y. Tian, D. G. Ashbrook, G. P. Cofer, J. J. Cook, J. C. Gee, A. Hall, K. Hornburg, Y. Qi, F.-C. Yeh, N. Wang, L. E. White, R. W. Williams, Merged magnetic resonance and light sheet microscopy of the whole mouse brain. Proc. Natl. Acad. Sci. 120, e2218617120 (2023). 37068254 10.1073/pnas.2218617120 PMC10151475

[11] D. K. Jones, *Diffusion MRI* (Oxford Univ. Press, 2010).

[12] D. L. Bihan, Looking into the functional architecture of the brain with diffusion MRI. Nat. Rev. Neurosci. 4, 469–480 (2003). 12778119 10.1038/nrn1119

[13] H. Lundell, C. Najac, M. Bulk, H. E. Kan, A. G. Webb, I. Ronen, Compartmental diffusion and microstructural properties of human brain gray and white matter studied with double diffusion encoding magnetic resonance spectroscopy of metabolites and water. Neuroimage 234, 117981 (2021). 33757904 10.1016/j.neuroimage.2021.117981 PMC8204266

[14] D. S. Novikov, E. Fieremans, S. N. Jespersen, V. G. Kiselev, Quantifying brain microstructure with diffusion MRI: Theory and parameter estimation. NMR Biomed. 32, e3998 (2019). 30321478 10.1002/nbm.3998 PMC6481929

[15] P. T. Callaghan, C. D. Eccles, Y. Xia, NMR microscopy of dynamic displacements: k-space and q-space imaging. J. Phys. E 21, 820–822 (1988).

[16] S. Lasǐ, H. Lundell, D. Topgaard, T. B. Dyrby, Effects of imaging gradients in sequences with varying longitudinal storage time—Case of diffusion exchange imaging. Mang. Res. Med. 79, 2228–2235 (2018). 10.1002/mrm.26856 28758240

[17] G. Balasubramanian, I. Y. Chan, R. Kolesov, M. Al-Hmoud, J. Tisler, C. Shin, C. Kim, A. Wojcik, P. R. Hemmer, A. Krueger, T. Hanke, A. Leitenstorfer, R. Bratschitsch, F. Jelezko, J. Wrachtrup, Nanoscale imaging magnetometry with diamond spins under ambient conditions. Nature 455, 648–651 (2008). 18833276 10.1038/nature07278

[18] J. R. Maze, P. L. Stanwix, J. S. Hodges, S. Hong, J. M. Taylor, P. Cappellaro, L. Jiang, M. V. Gurudev Dutt, E. Togan, A. S. Zibrov, A. Yacoby, R. L. Walsworth, M. D. Lukin, Nanoscale magnetic sensing with an individual electronic spin in diamond. Nature 455, 644–647 (2008). 18833275 10.1038/nature07279

[19] C. Müller, X. Kong, J.-M. Cai, K. Melentijević, A. Stacey, M. Markham, D. Twitchen, J. Isoya, S. Pezzagna, J. Meijer, J. F. Du, M. B. Plenio, B. Naydenov, L. P. McGuinness, F. Jelezko, Nuclear magnetic resonance spectroscopy with single spin sensitivity. Nat. Comun. 5, 4703 (2014). 10.1038/ncomms5703 PMC4143926 25146503

[20] A. O. Sushkov, I. Lovchinsky, N. Chisholm, R. L. Walsworth, H. Park, M. D. Lukin, Magnetic resonance detection of individual proton spins using quantum reporters. Phys. Rev. Lett. 113, 197601 (2014). 25415924 10.1103/PhysRevLett.113.197601

[21] T. Staudacher, F. Shi, S. Pezzagna, J. Meijer, J. Du, C. A. Meriles, F. Reinhard, J. Wrachtrup, Nuclear magnetic resonance spectroscopy on a (5-nanometer)^3^ sample volume. Science 339, 561–563 (2013). 23372009 10.1126/science.1231675

[22] H. J. Mamin, M. Kim, M. H. Sherwood, C. T. Rettner, K. Ohno, D. D. Awschalom, D. Rugar, Nanoscale nuclear magnetic resonance with a nitrogen-vacancy spin sensor. Science 339, 557–560 (2013). 23372008 10.1126/science.1231540

[23] K. S. Liu, A. Henning, M. W. Heindl, R. D. Allert, J. D. Bartl, I. D. Sharp, R. Rizzato, D. B. Bucher, Surface nmr using quantum sensors in diamond. PNAS 119, e2111607119 (2022). 35082146 10.1073/pnas.2111607119 PMC8812553

[24] D. R. Glenn, D. B. Bucher, J. Lee, M. D. Lukin, H. Park, R. L. Walsworth, High-resolution magnetic resonance spectroscopy using a solid-state spin sensor. Nature 555, 351–354 (2018). 29542693 10.1038/nature25781

[25] N. Arunkumar, D. B. Bucher, M. J. Turner, P. T. Hon, D. Glenn, S. Lehmkuhl, M. D. Lukin, H. Park, M. S. Rosen, T. Theis, R. L. Walsworth, Micron-scale NV-NMR spectroscopy with signal amplification by reversible exchange. PRX Quantum 2, 010305 (2021).

[26] J. Smits, J. T. Damron, P. Kehayias, A. F. Mc Dowell, N. Mosavian, I. Fescenko, N. Ristoff, A. Laraoui, A. Jarmola, V. M. Acosta, Two-dimensional nuclear magnetic resonance spectroscopy with a microfluidic diamond quantum sensor. Sci. Adv. 5, eaaw7895 (2019). 31360769 10.1126/sciadv.aaw7895 PMC6660203

[27] D. B. Bucher, D. R. Glenn, H. Park, M. D. Lukin, R. L. Walsworth, Hyperpolarization-enhanced NMR spectroscopy with femtomole sensitivity using quantum defects in diamond. Phys. Rev. X 10, 021053 (2020).

[28] R. D. Allert, K. D. Briegel, D. B. Bucher, Advances in nano- and microscale NMR spectroscopy using diamond quantum sensors. Chem. Commun. 58, 8165–8181 (2022). 10.1039/d2cc01546c PMC9301930 35796253

[29] R. D. Allert, F. Bruckmaier, N. R. Neuling, F. A. Freire-Moschovitis, K. S. Liu, C. Schrepel, P. Schätzle, P. Knittel, M. Hermans, D. B. Bucher, Microfluidic quantum sensing platform for lab-on-a-chip applications. Lab Chip 22, 4831–4840 (2022). 36398977 10.1039/d2lc00874b

[30] F. Bruckmaier, K. D. Briegel, D. B. Bucher, Geometry dependence of micron-scale NMR signals on NV-diamond chips. J. Magn. Reson. Open 8-9, 100023 (2021).

[31] T. Wolf, P. Neumann, K. Nakamura, H. Sumiya, T. Ohshima, J. Isoya, J. Wrachtrup, Subpicotesla diamond magnetometry. Phys. Rev. X 5, 041001 (2015).

[32] N. Arunkumar, K. S. Olsson, J. T. Oon, C. Hart, D. B. Bucher, D. Glenn, M. D. Lukin, H. Park, D. Ham, R. L. Walsworth, Quantum logic enhanced sensing in solid-state spin ensembles. arXiv:2203.12501 (23 March 2022). 10.1103/PhysRevLett.131.100801 37739376

[33] P. Amrein, F. Jia, M. Zaitsev, S. Littin, CoilGen: Open-source MR coil layout generator. Magn. Reson. Med. 88, 1465–1479 (2022). 35526237 10.1002/mrm.29294

[34] J.-P. Tetienne, L. Rondin, P. Spinicelli, M. Chipaux, T. Debuisschert, J.-F. Roch, V. Jacques, Magnetic-field-dependent photodynamics of single NV defects in diamond: An application to qualitative all-optical magnetic imaging. N. J. Phys. 14, 103033 (2012).

[35] P. Amrein, F. Bruckmaier, F. Jia, D. B. Bucher, M. Zaitsev, S. Littin, Optimal bi-planar gradient coil configurations for diamond nitrogen-vacancy based diffusion-weighted NMR experiments. Magn Reson Mater Phy. 10.1007/s10334-023-01111-0 (2023). PMC10667408 37578612

[36] E. V. Levine, M. J. Turner, P. Kehayias, C. A. Hart, N. Langellier, R. Trubko, D. R. Glenn, R. R. Fu, L. Ronald, Walsworth., Principles and techniques of the quantum diamond microscope. Nanophotonics 8, 1945–1973 (2019).

[37] P. B. Kingsley, Introduction to diffusion tensor imaging mathematics: Part III. Tensor calculation, noise, simulations, and optimization. Magn. Reson. A 28A, 155–179 (2006).

[38] P. T. Callaghan, Y. Xia, Velocity and diffusion imaging in dynamic NMR microscopy. J. Magn. Reson. 91, 326–352 (1991).

[39] N. H. Williamson, M. E. Komlosh, D. Benjamini, P. J. Basser, Limits to flow detection in phase contrast MRI. J. Magn. Reson. Open 2-3, 100004 (2020). 33345200 10.1016/j.jmro.2020.100004 PMC7745993

[40] R. Mills, Self-diffusion in normal and heavy water in the range 1-45.deg. J. Phys. Chem. 77, 685–688 (1973).

[41] K. R. Harris, L. A. Woolf, Pressure and temperature dependence of the self diffusion coefficient of water and oxygen-18 water. J. Chem. Soc. Faraday Trans. 1 76, 377–385 (1980).

[42] P. S. Tofts, D. Lloyd, C. A. Clark, G. J. Barker, G. J. M. Parker, P. McConville, C. Baldock, J. M. Pope, Test liquids for quantitative MRI measurements of self-diffusion coefficient in vivo. Magn. Reson. Med. 43, 368–374 (2000). 10725879 10.1002/(sici)1522-2594(200003)43:3<368::aid-mrm8>3.0.co;2-b

[43] F. Wagner, F. B. Laun, T. A. Kuder, A. Mlynarska, F. Maier, J. Faust, K. Demberg, L. Lindemann, B. Rivkin, A. M. Nagel, M. E. Ladd, K. Maier-Hein, S. Bickelhaupt, M. Bach, Temperature and concentration calibration of aqueous polyvinylpyrrolidone (PVP) solutions for isotropic diffusion MRI phantoms. PLOS ONE 12, e0179276 (2017). 28628638 10.1371/journal.pone.0179276 PMC5476261

[44] J. Eills, W. Hale, M. Utz, Synergies between hyperpolarized NMR and microfluidics: A review. Prog. Nucl. MAgn. Reson. Scpectros. 128, 44–69 (2022). 10.1016/j.pnmrs.2021.09.001 35282869

[45] R. Kimmich, W. Unrath, G. Schnur, E. Rommel, NMR measurement of small self-diffusion coefficients in the fringe field of superconducting magnets. J. Magn. Reson. 91, 136–140 (1991).

[46] P. T. Callaghan, M. E. Komlosh, M. Nyden, High magnetic field gradient PGSE NMR in the presence of a large polarizing field. J. Magn. Reson. 133, 177–182 (1998). 9654483 10.1006/jmre.1998.1424

[47] H. Zhang, K. Arai, C. Belthangady, J.-C. Jaskula, R. L. Walsworth, Selective addressing of solid-state spins at the nanoscale via magnetic resonance frequency encoding. npj Quant. Inf. 3, 31 (2017).

[48] K. Arai, C. Belthangady, H. Zhang, N. Bar-Gill, S. J. DeVience, P. Cappellaro, A. Yacoby, R. L. Walsworth, Fourier magnetic imaging with nanoscale resolution and compressed sensing speed-up using electronic spins in diamond. Nat. Nanotechnol. 10, 859–864 (2015). 26258549 10.1038/nnano.2015.171

[49] F. Szczepankiewicz, C.-F. Westin, M. Nilsson, Maxwell-compensated design of asymmetric gradient waveforms for tensor-valued diffusion encoding. Magn. Reson. Med. 82, 1424–1437 (2019). 31148245 10.1002/mrm.27828 PMC6626569

[50] H. H. Heenen, C. Scheurer, K. Reuter, Implications of occupational disorder on ion mobility in li−4*ti*_5_*o*_12_ battery materials. Nano Lett. 17, 3884–3888 (2017). 28514174 10.1021/acs.nanolett.7b01400

[51] P. Benedek, O. K. Forslund, E. Nocerino, N. Yazdani, N. Matsubara, Y. Sassa, F. Jurànyi, M. Medarde, M. Telling, M. Månsson, V. Wood, Quantifying diffusion through interfaces of lithium-ion battery active materials. ACS Appl. Mater. Interf. 12, 16243–16249 (2020). 10.1021/acsami.9b21470 32163263

[52] C. M. W. Tax, F. Szczepankiewicz, M. Nilsson, D. K. Jones, The dot-compartment revealed? diffusion MRI with ultra-strong gradients and spherical tensor encoding in the living human brain. Neuroimage 210, 116534 (2020). 31931157 10.1016/j.neuroimage.2020.116534 PMC7429990

[53] I. O. Jelescu, L. Ciobanu, F. Geffroy, P. Marquet, D. L. Bihan, Effects of hypotonic stress and ouabain on the apparent diffusion coefficient of water at cellular and tissue levels in *Aplysia*. NMR Biomed. 27, 280–290 (2014). 24403001 10.1002/nbm.3061

[54] D. Wu, J. Xu, M. T. McMahon, P. C. M. van Zijl, S. Mori, F. J. Northington, J. Zhang, In vivo high-resolution diffusion tensor imaging of the mouse brain. Neuroimage 83, 18–26 (2013). 23769916 10.1016/j.neuroimage.2013.06.012 PMC3797856

[55] P. Schätzle, P. Reinke, D. Herrling, A. Götze, L. Lindner, J. Jeske, L. Kirste, P. Knittel, A chemical vapor deposition diamond reactor for controlled thin-film growth with sharp layer interfaces. Phys. Status Solidi 220, 2200351 (2022).

[56] D. B. Bucher, D. P. L. Aude Craik, M. P. Backlund, M. J. Turner, O. B. Dor, D. R. Glenn, R. L. Walsworth, Quantum diamond spectrometer for nanoscale NMR and ESR spectroscopy. Nat. Protoc. 14, 2707–2747 (2019). 31451784 10.1038/s41596-019-0201-3

[57] R. A. Lemdiasov, R. Ludwig, A stream function method for gradient coil design. Concepts Magn. Reson. Part B Magn. Reson. Eng. 26B, 67–80 (2005).

[58] G. T. Genov, D. Schraft, N. V. Vitanov, T. Halfmann, Arbitrarily accurate pulse sequences for robust dynamical decoupling. Phys. Rev. Lett. 118, 133202 (2017). 28409941 10.1103/PhysRevLett.118.133202

[59] T. A. A. Cartlidge, T. B. R. Robertson, M. Utz, G. Pileio, Theory and simulation framework for the relaxation of nuclear spin order in porous media. J. Phys. Chem. B 126, 6536–6546 (2022). 35976731 10.1021/acs.jpcb.2c03575 PMC9442653

[60] N. Neuling, R. D. Allert, D. B. Bucher, Prospects of single-cell NMR spectroscopy with quantum sensors. Current Opinion in Biotechnology 83, 102975 (2023). 37573624 10.1016/j.copbio.2023.102975

[61] C. L. Degen, F. Reinhard, P. Cappellaro, Quantum sensing. Rev. Mod. Phys. 89, 035002 (2017).

[62] R. Rizzato, F. Bruckmaier, K. S. Liu, S. J. Glaser, D. B. Bucher, Polarization transfer from optically-pumped NV center ensembles to multinuclear spin baths. Phys. Rev. Applied 17, 024067 (2022).

[63] J. F. Barry, J. M. Schloss, E. Bauch, M. J. Turner, C. A. Hart, L. M. Pham, R. L. Walsworth, Sensitivity optimization for NV-diamond magnetometry. Rev. Mod. Phys. 92, 015004 (2020).

[64] M. W. Doherty, N. B. Manson, P. Delaney, F. Jelezko, J. Wrachtrup, L. C. L. Hollenberg, The nitrogen-vacancy colour centre in diamond. Phys. Rep. 528, 1–45 (2013).

[65] H. Y. Carr, E. M. Purcell, Effects of diffusion on free precession in nuclear magnetic resonance experiments. Phys. Rev. 94, 630–638 (1954).

[66] F. Reinhard, *Nanoscale Sensing and Quantum Coherence* (IOS Press, 2019).

[67] P. P. Mitra, P. N. Sen, L. M. Schwartz, Short-time behavior of the diffusion coefficient as a geometrical probe of porous media. Phys. Rev. B 47, 8565–8574 (1993). 10.1103/physrevb.47.8565 10004895

[68] J. E. Tanner, E. O. Stejskal, Restricted self-diffusion of protons in colloidal systems by the pulsed-gradient, spin-echo method. J. Chem. Phys. 49, 1768–1777 (1968).

[69] A. Kusumi, Y. Sako, M. Yamamoto, Confined lateral diffusion of membrane receptors as studied by single particle tracking (nanovid microscopy). effects of calcium-induced differentiation in cultured epithelial cells. Biophys. J. 65, 2021–2040 (1993). 8298032 10.1016/S0006-3495(93)81253-0 PMC1225938

[70] M. Fujiwara, Y. Shikano, Diamond quantum thermometry: From foundations to applications. Nanotechnology 32, 482002 (2021). 10.1088/1361-6528/ac1fb1 34416739

