# Supplementary material for: Imaging local diffusion in microstructures using NV-based pulsed field gradient NMR
Source: Sci Adv. 2023 Aug 18;9(33):eadh3484. doi: 10.1126/sciadv.adh3484 (PMC10438442; doi:10.1126/sciadv.adh3484)
Supplement: Supplementary file 1 — Sections S1 to S10 Figs. S1 to S15 References [file sciadv.adh3484_sm.pdf]

Science Advances
AAAS

Supplementary Materials for

Imaging local diffusion in microstructures using NV-based pulsed field gradient NMR

Fleming Bruckmaier *et al.*

Corresponding author: Dominik B. Bucher, dominik.bucher@tum.de

Sci. Adv. **9**, eadh3484 (2023)
DOI: 10.1126/sciadv.adh3484

This PDF file includes:

Sections S1 to S10
Figs. S1 to S15
References

1 Short summary of the working principle of the NV center

The NV center is a point defect in the diamond crystal lattice (Fig. S1b), where one of the carbon atoms is replaced by a nitrogen atom and an adjacent lattice site is left vacant, resulting in a C_{3v} symmetry. The NV center can exists in different charge states, but only the negatively charged NV^- state is applicable for quantum sensing. Therefore, we will refer to it as "NV center".

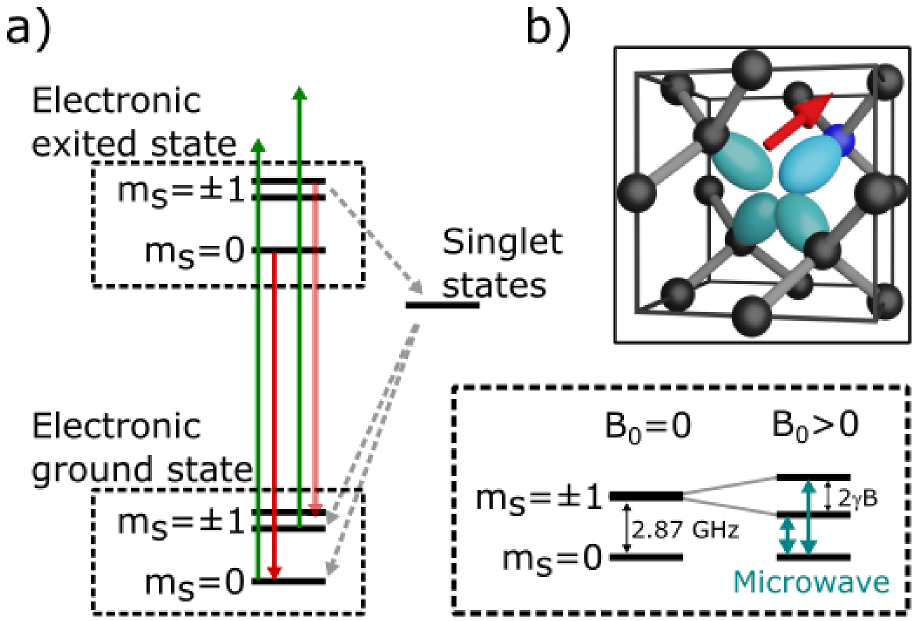

Figure S1: **The energy level diagram and structure of the NV center in diamond**

The NV center's electronic ground and excited states are triplet states, located within the diamond band-gap (*64*). The corresponding $\mathrm{m_s} = 0$ and $\mathrm{m_s} = \pm 1$ in the electronic ground state are separated by a zero-field splitting of $\mathrm{D} = 2.87$ GHz, whereas the $\mathrm{m_s} = \pm 1$ degeneracy can be lifted according to the Zeeman splitting $\Delta E = 2\gamma B$, where $\gamma \approx 28$ GHz/T is the electron's gyromagnetic ratio. The NV-center can be excited with green light (green arrows, Fig. S1a) and will decay back into its respective electronic ground state by emitting red fluorescence (red arrows, Fig. S1a). The $\mathrm{m_s} = \pm 1$ spin states have an additional, non-radiative decay channel via

singlet states, which in turn can decay into the $\mathrm{m_s}$ = $\pm$ 1 as well as $\mathrm{m_s}$ = 0 states. This leads to i) the possibility of initializing the NV center in the $\mathrm{m_s}$ = 0 state by optical pumping, and ii) to a spin-state dependent fluorescence, which allows to read out the NV-center's quantum state optically. Lastly, the magnetic sublevels can be coherently addressed with microwave pulses (turquoise arrows, Fig. S1a).

2 NV-AC magnetometry using CASR

For most AC-sensing applications (such as the detection of NMR signals) the NV-center is dynamically decoupled from the environment by a train of π-pulses with a spacing 2τ. In the simplest case, this is the Carr-Purcell-Meiboom-Gill (CPMG) sequence (*61, 65*). We use one of its variations called universally robust dynamic decoupling (UDD) (*58*), as it is more robust against pulse errors. These pulse sequences act as a narrow-band frequency filter around $f_{sense} = \frac{1}{4\tau}$ (Fig. S2): Magnetic field frequencies not matching this condition will be suppressed by the spin echoes of the dynamic decoupling pulse sequence. Frequencies matching this condition will lead to a phase accumulation on the NV-center's spin state (related to electron spin echo envelope modulation "ESEEM" in conventional spin resonance experiments), which can be read out optically and is used herein for the detection of NMR signals. In our experiments we use a modified version called coherently averaged synchronized readout (CASR) (*24*). It relies on the fact that the signal detected by dynamical decoupling sequences is sensitive to the relative phase of the ac signal (NMR signal) and the pulse sequence (i.e., the train of π pulses). If the target ac signal is asymmetric around the π-pulse, then the phase accumulation is maximal. This is due to the simultaneous "change in sign" of both the oscillating magnetic field and the NV-center's spin. If in turn the ac signal is symmetric around the π-pulses, no phase will be acquired and the quantum state of the NV is not influenced. If the period of the sensing frequency is an integer multiple of each measurement repetition, the phase relation will be

constant between consecutive readouts. However, if the frequency is not matched as depicted in Fig. S2, we observe aliasing and the fluorescence readout will oscillate at the difference frequency. The advantage of this pulse sequence is, that NMR signals can be detected with high spectral resolution, since the only limitation is the lifetime of the NMR-active nuclei under investigation (assuming insignificant back-action between the NV and samples spins). A more detailed description of this process can be found in the literature (*24, 56, 63, 66*).

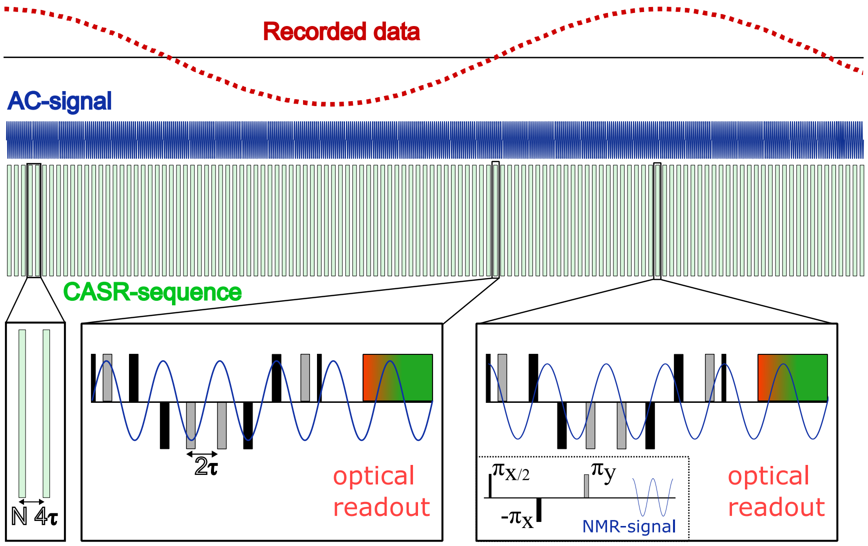

Figure S2: **Detecting NMR signals using the CASR pulse sequence.** A sketch of CASR's working principle. The spacing of the individual measurement blocks (dynamic decoupling sequence and the optical readout pulse) is a multiple of a frequency close to the target AC-signal (blue). The difference in the initial phase of the AC-signal relative to the start of each individual block (green rectangles), leads to an oscillation of the phase accumulation of the NV-center's spin state over consecutive measurements. This, in turn, causes an oscillation of the NV fluorescence intensity in the recorded data (red). This is illustrated by the two insets in the bottom right, the first having a relative phase leading to minimal signal amplitude, the second leading to maximum signal amplitude.

3 Experimental setup, figures

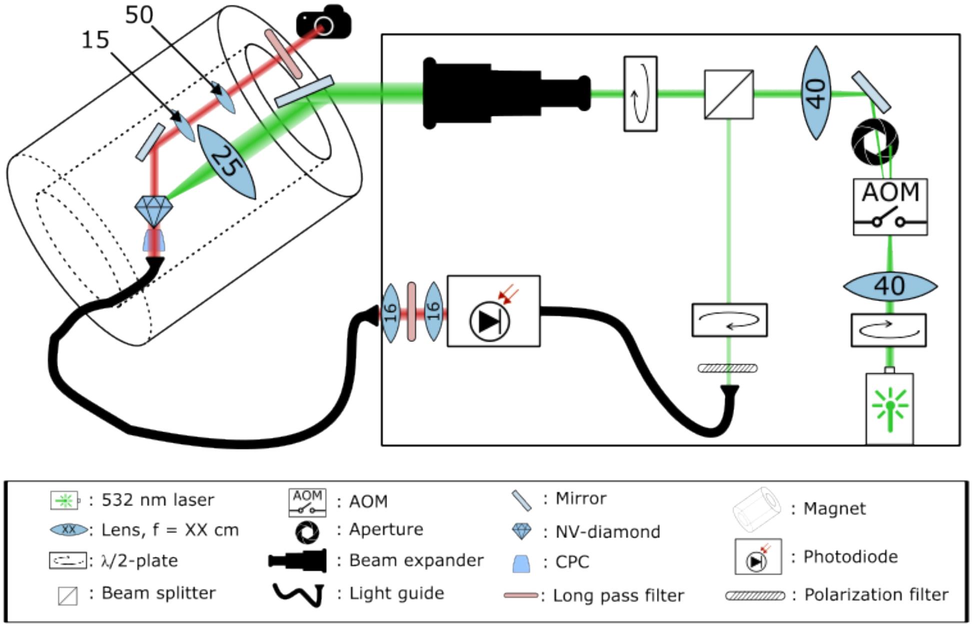

Figure S3: **Experimental setup: Optics.** The numbers associated with each of the lenses indicates the respective focal length in cm.

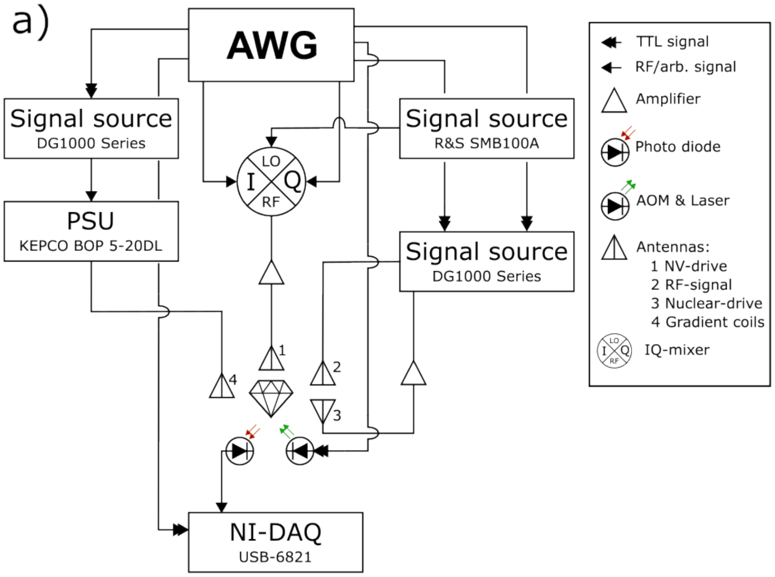

Figure S4: **Experimental setup: Electronics**.

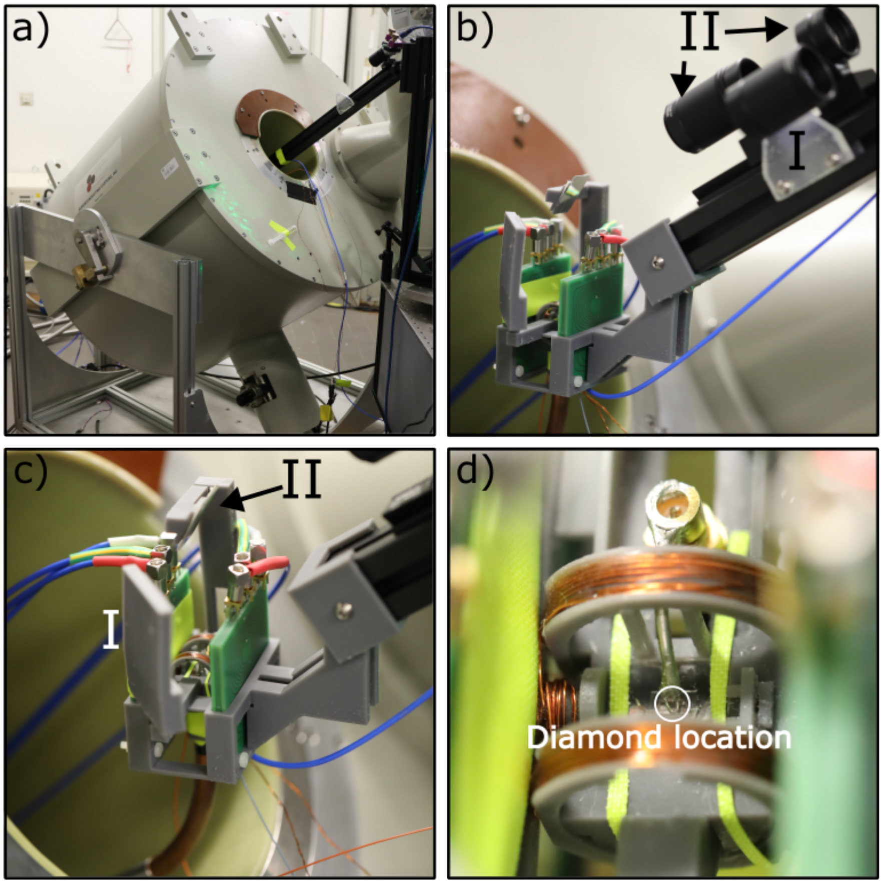

Figure S5: **Photos of the experimental setup.** **a)** Superconducting magnet mounted on a tiltable and rotating platform for magnetic field alignment. The probe head is mounted on a aluminum rod. **b)** Photo of the probe head. The two lenses (II) are used for optical imaging, while the excitation laser is focused on the diamond chip using lens I. **c)** The 3D-printed sample holder. The excitation laser is reflected onto the diamond chip using a mirror at I, while the diamond is imaged using mirror II. The magnetic field gradient coils are stacked into the holder (the dark green PCBs). **d)** A zoom-in on the diamond chip. The copper-coils in Helmholtz-geometry are used for driving the sample nuclear spins. A small RF coil (right) is used for calibrating the experiment. A shortened coaxial cable used for driving the NV centers is mounted on the microfluidic chip (center).

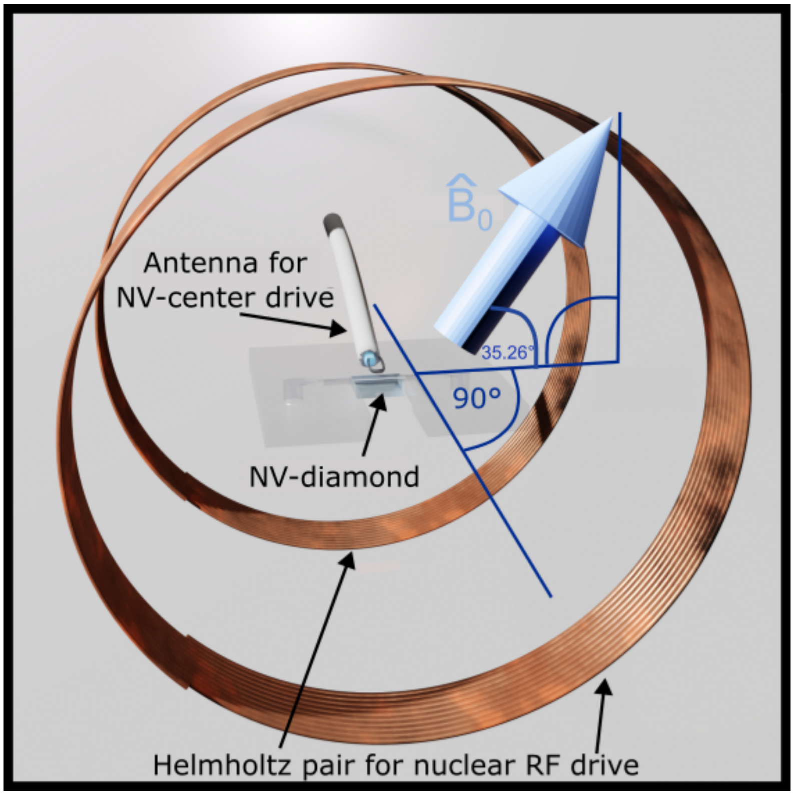

Figure S6: **Relative position and orientations of the core components.**

4 PGSE in a microfluidic channel

In the following section, we outline the basic theory of restricted diffusion and discuss the modeling of our diffusion experiments. The theory behind sample-diffusion is well understood (*6*) including the case of restricted diffusion on the microscale (*67–69*).

A Analytical treatment and numerical simulation of restricted diffusion on short time scales

For a given free diffusion constant D_0, the distance traveled per molecule is proportional to $\sqrt{D_0 t}$. Following Kusumi *et al.* (*69*), the time-dependent, apparent diffusion coefficient $ADC(t)$ in one dimension, *e.g.* between two parallel plates, can be calculated according to:

$$ADC\left(t\right) = \frac{\left\langle \left(x_0' - x'(t)\right)^2 \right\rangle}{2t} = \frac{L^2}{6} - 16\frac{L^2}{\pi^4}\sum_{n=1}^{\infty}\frac{1}{\left(2n-1\right)^4}\ \exp(-\left(\pi\frac{2n-1}{L}\right)^2 D_0 t)\ . \quad \text{(S1)}$$

where L is the distance between the two plates x' and $\left\langle \left(x_0' - x'(t)\right)^2 \right\rangle$ is the mean squared particle displacement. These results are identical to the diffusion between two parallel walls of a cuboid microfluidic channel, and represent the eigenvalues of the diffusion tensor. In the case of the microfluidic channel this would be the basis, in which each of the basis vectors is orthogonal to one set of the channel-walls. The measurement basis in our experiments follows the NMR-convention ($B_0||\hat{z}$), which is equivalent to a rotation of the previous coordinate system around $\hat{x}'$ by $\phi \approx 35.26°$, with $\hat{x}' = \hat{x}$, $\hat{y}' \rightarrow \hat{y}$ and $\hat{z}' \rightarrow \hat{z}$. This leads to the effective diffusion constants of:

$$\begin{aligned} ADC_{\hat{x}}\left(t\right) &= ADC_{\hat{x}'}\left(t\right) \\ ADC_{\hat{y}}\left(t\right) &= \cos^2(\phi) ADC_{\hat{y}'}\left(t\right) + \sin^2(\phi) ADC_{\hat{z}'}\left(t\right)\ . \\ ADC_{\hat{z}}\left(t\right) &= \sin^2(\phi) ADC_{\hat{y}'}\left(t\right) + \cos^2(\phi) ADC_{\hat{z}'}\left(t\right) \end{aligned} \quad \text{(S2)}$$

The same results can be obtained by numerical simulations using a random walk in a sample volume of the size of our microfluidic channel. At each time step Δt, each sample point is moved by a randomly Gaussian distributed amount. If it would leave the sample volume, it is reflected back inwards. From this we calculated the mean-squared displacement and in turn the effective diffusion constant along each direction, according to:

$$ADC_{\hat{x}_i}(t) = \frac{\left\langle \left[\hat{x}_i \cdot \left(\vec{r}_0 - \vec{r}(t)\right)\right]^2 \right\rangle}{2t}\ . \quad \text{(S3)}$$

A comparison of the analytical and numerically determined effective diffusion constant is shown in Fig. S7.

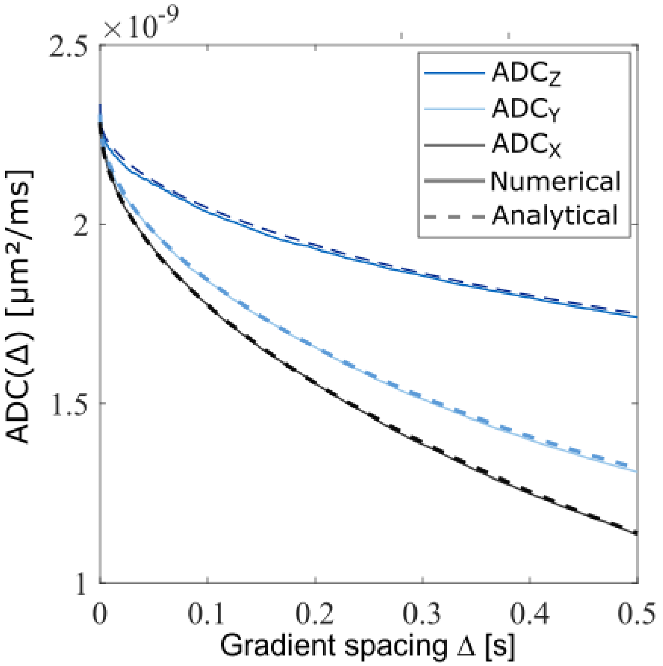

Figure S7: **Simulated ADCs in a microfluidic channel.** The apparent diffusion coefficient as a function of gradient pulse spacing Δ within our microfluidic chip (100 μm in $\hat{x}$ direction, 80 μm in $\hat{y}$' direction and 2000 μm in $\hat{z}$' direction,) along the three directions measured. Analytical values are displayed as dotted lines, numerical values as full lines.

For a comparison with the experimental data of the microstructure in Fig.5 in the main manuscript, we performed simulations in this geometry, seen in Fig. S8. For each pixel 1000 particles were placed at the respective coordinates, evenly spaced between the bottom and top of the microfluidic channel. The particles underwent a Gaussian random walk in 101 equally spaced steps for 0.1 s. The ADC was then calculated according to equation S3.

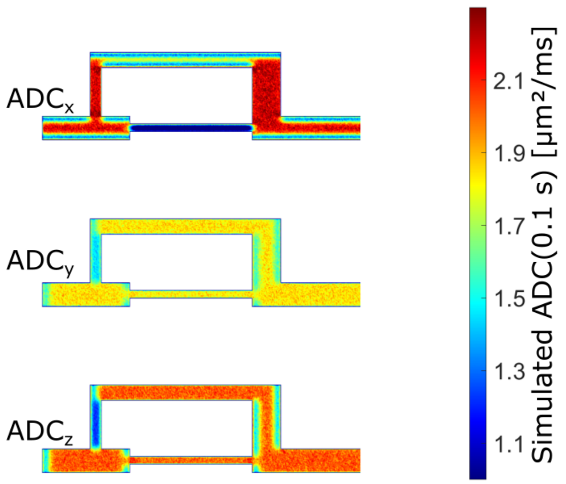

Figure S8: **Simulated ADC in a microstructure.** The spatial resolved ADC in the microfluidic channel used for the experiments shown in Fig. 5 in the main document, assuming a diffusion coefficient of $D = 2.2 \times\ \mu\text{m}^2$/ms.

B Influence of the NV center's sensitivity map on diffusion measurements

In this paragraph, we discuss the possible influence of the strongly geometry-dependent NV-NMR detection on the PGSE-experiments (*30*). The field of a magnetic dipole, *e.g.* a nuclear spin in our sample, is described by:

$$\vec{B}_{\text{spin}}(\vec{r},t) = \frac{\mu_0}{4\pi}\left[\frac{3\hat{r}\,(\hat{r}\cdot\vec{m}(t)) - \vec{m}(t)}{r^3}\right] , \qquad \text{(S4)}$$

where μ_0 is the magnetic permeability, $\hat{r}$ is the direction from the sample spin to the NV center, and $r = |\vec{r}\,|$ is the distance between the two. $\vec{m}(t)$ is the magnetic moment of the precessing nuclear spin. NV-NMR typically measures the parallel magnetic field component, via the Zeeman shift of the NV center's $|\pm1\rangle$ levels. Projecting $\vec{B}_{\text{spin}}(\vec{r},t)$ onto $\vec{B}_0$, we obtain:

$$B_{\text{spin}||}(\vec{r},t) = \frac{\mu_0}{4\pi}\left[\frac{3(\hat{r}\cdot\hat{B}_0)\,(\hat{r}\cdot\vec{m}(t)) - (\vec{m}(t)\cdot\hat{B}_0)}{r^3}\right] . \qquad \text{(S5)}$$

Since the magnetization of the precessing spins are orthogonal to $\hat{B}_0$, after a $\pi/2$-pulse,

$(\vec{m}(t) \cdot \hat{B}_0) = 0$, leading to a magnetic field contribution $\vec{B}_{\text{spin}}(\vec{r}, t)$ of a sample-spin at position $\vec{r}$:

$$B_{\text{spin}||}(\vec{r}, t) = \frac{\mu_0}{4\pi} \left[\frac{3(\hat{r} \cdot \hat{B}_0)\,(\hat{r} \cdot \vec{m}(t))}{r^3} \right] . \tag{S6}$$

A visualization of this sensitivity map can be found in Fig. S9 a) and b).

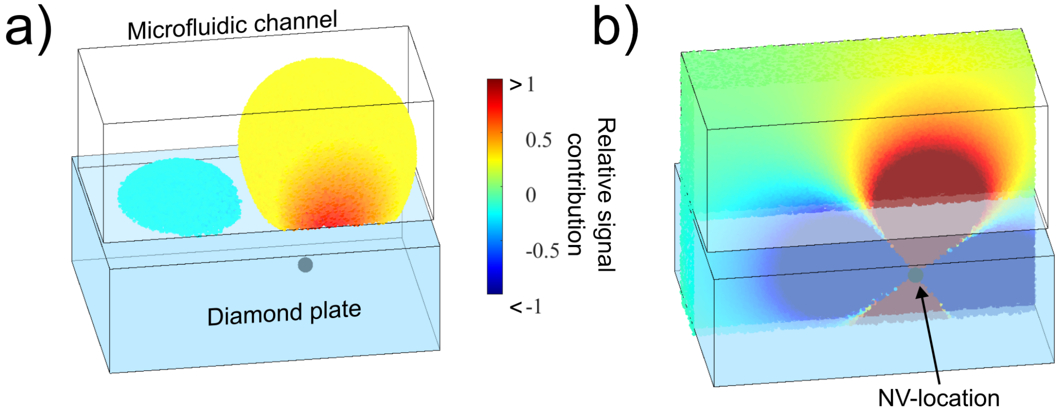

Figure S9: **Sensitivity map of our NV-NMR experiment.** Calculated relative signal contribution of water molecules in our microfluidic channel for a NV center at the indicated location. Red areas contribute positively to the signal and blue areas negatively. **a)** Only the areas with the highest magnitude contribution (top 40 % of simulated volume) are displayed. **b)** A cross-section through the channel for a clearer visualization of the sensitivity map. The sensitivity map within the diamond is also displayed, despite the absence of any sample nuclei in this volume.

In particular the two terms $1/r^3$ and $(\hat{r} \cdot \hat{B}_0)$ can have a strong impact on the directional sensitivity of our NV centers. Since the time independent contribution to the signal amplitude is of importance, we integrate over one period of sample spin oscillation T and arrive at a relative position dependence c of each individual spin, according to:

$$c(\vec{r}) = \int_0^T |\vec{B}_{\text{spin}||}(\vec{r}, t)|\, dt\, \frac{1}{C} , \tag{S7}$$

where C is chosen such that

$$\int c(\vec{r})\, dV_{\text{sample}} = 1 . \tag{S8}$$

We note that, for our purposes, this integral is convergent as a function of sample volume (*30*), therefore the bulk of the signal stems from a local area close to the diamond surface, whose size is of the same order of magnitude as the depth of the NV-center. For highly anisotropic samples, an additional contribution to the signal can appear, since sample areas with positive and negative signal contributions at the NV-location might not cancel out. Nevertheless, even in a worst-case scenario, the total signal from dipoles with a distance from the NV center much bigger than the NV center's depth below the diamond surface will only increase logarithmically as a function of sample volume (*30*). If the NV-layer is chosen such that the layer thickness is not several orders of magnitude larger than the smallest sample dimension, this additional signal can probably be considered to be insignificant.

Finally, we can define a position-weighted diffusion coefficient in each direction according to:

$$D_{weight,\hat{x}_i}(t) = \frac{1}{2t} \int c(\vec{r}) \left[\hat{x}_i \cdot (\vec{r}_0 - \vec{r}(t))\right]^2 \, dV_{\text{sample}} \, . \tag{S9}$$

The diffusion coefficient weighted by the coupling to an ensemble of NVs, distributed over a cylindrical volume of 45 μm diameter and 50 μm depth was simulated. The resulting difference between the weighted diffusion coefficients with respect to their analytical solutions from equation S2 is plotted in Fig. S10. The difference between the weighted simulation and analytical results stays below 1 % of the analytical value for $\Delta = 0.5$ s in the $\hat{y}$ direction and below 1 % for both $\hat{x}$ and $\hat{z}$ for more than $\Delta = 0.5$ s. In the interpretation of our data, this difference is therefore treated as negligible.

We also note that, although a significant fraction of the molecules may diffuse out of the detection volume and be replaced by other molecules after the second gradient pulse, this will not affect the recorded signal. We assume that both the $B0$ field and the RF field, driving the nuclear sample spins, are homogeneous over an area much larger than i) the size of the

laser spot, which defines the detection volume, and ii) the mean distance diffused during the measurement time. For example, the sample spins probed in the different locations in Fig. 5 in the main manuscript all have indistinguishable Rabi frequencies, despite their locations are several hundred micrometers apart. Assuming the diffusion constant and environment, i.e. local anisotropicity, of all spins entering and leaving the probed volume is the same, they should underlie the same Gaussian process and lead to the same signal attenuation, resulting in no net effect on the recorded diffusion constant. Therefore, the so-called "diffusion blurring" during the diffusion weighting with magnetic field gradients will not contribute to the reduction of the spatial resolution. Contrary to that, diffusion processes during the acquisition of the spin-echo from the sample will contribute with the conventional square-root dependence of the induced blurring on the acquisition time, which mandates using short data acquisition windows if high spatial resolution is desired.

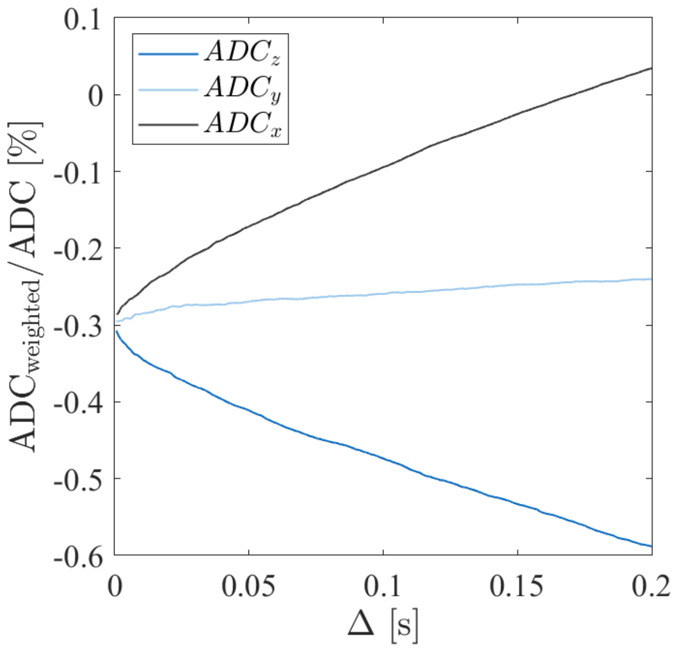

Figure S10: **Impact of NV center sensitivity map on diffusion measurements.** The difference in percent between the diffusion tensors weighted diagonal elements with respect to the unweighted analytical value as a function of gradient pulse spacing Δ.

5 Estimating the impact of a heterogeneous flow profile

The laminar flow in our microfluidic channel has a quadratic velocity distribution across the cross-section of the channel. In the case of a homogeneous sensitivity over the whole channel, the phase-shift measured will depend on the average velocity in the channel, which is set as constant at the flow pump and therefore independent of any velocity distribution. This means the only effect of a quadratic flow profile should be signal attenuation. However, the assumption of a spatially homogeneous sensitivity does not apply for NV-NMR (see section 4).

We used numerical simulations to analyze the effect of the interplay between the quadratic flow profile and the sensitivity map of the NV center (Fig. S11). The maximum velocity of the quadratic flow profile v_{max} relates to the set or average velocity v_{mean} as: $v_{max} = 3/2\ v_{mean}$.

Since the phase shift, $\delta\phi$ induced in the PGSE experiment is linear with the velocity, the flow profile was included in the simulation using a quadratic phase shift over the channel's cross section with a maximum phase shift $\delta\phi_{max} = 3/2\ \delta\phi_{mean}$. For 201 linearly spaced points between $\delta\phi_{mean} = 0$ and $\delta\phi_{mean} = 2\pi$ the corresponding NV-NMR signal phase $\delta\phi_{simulated}$ was simulated for $\Delta = 0.3$ s. As shown in Fig. S11, the simulated signal phase shift $\delta\phi_{simulated}$ initially deviates slightly from the expected linear dependence on the mean sample phase shift $\delta\phi_{mean}$, though for $\delta\phi_{mean} 1.5\,\pi$ the deviation becomes significant. In the experiments performed in the main manuscript, the expected mean sample phase shift does not exceed $\delta\phi_{mean} \approx 1.27\,\pi$. The discrepancy between mean sample phase shift and signal phase shift is corrected in the data displayed in the main manuscript by fitting a polynomial of order 4 to $\delta\phi_{mean}(\delta\phi_{simulated})$. This fit maps the recorded measurement value to the expected mean velocity according to our numerical simulation.

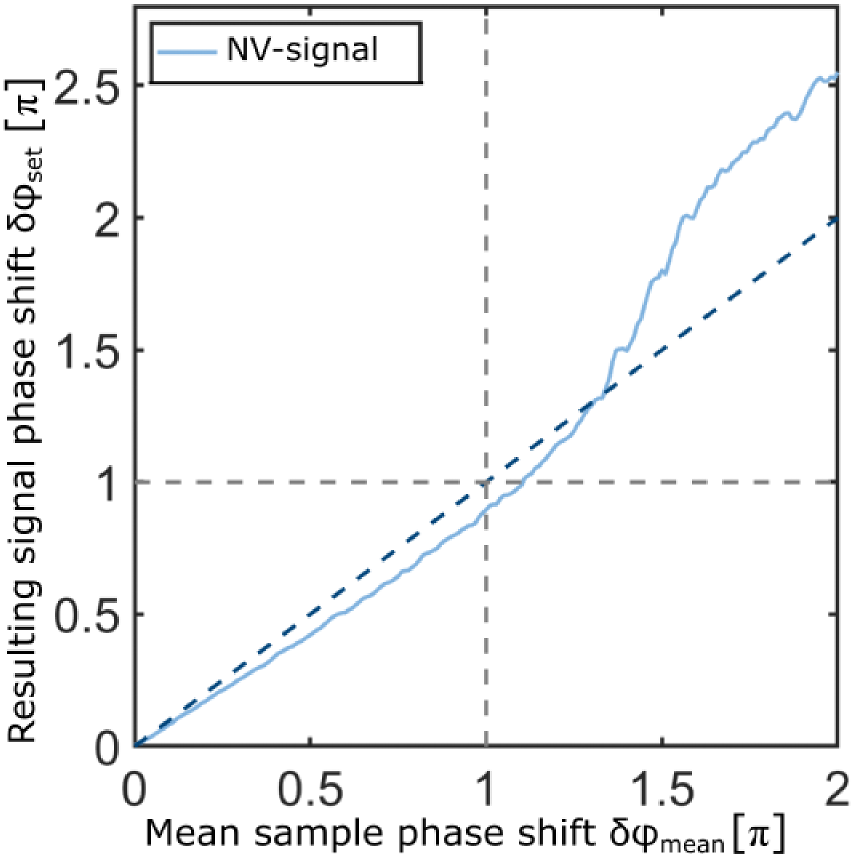

Figure S11: **Simulated phase shift for velocimetry experiments.** The extracted NV-NMR signal phase shift is plotted as a function of the set, mean phase shift (light blue). A linear dependency of $\delta\phi_{mean}\ \alpha\ \delta\phi_{set}$ is depicted with a dashed, dark blue line. Dashed, grey guiding lines were added for both $\delta\phi_{set} = \pi$ and $\delta\phi_{mean} = \pi$.

6 Outlook and feasibility of high spatial resolution PGSE NV-NMR

Increasing the spatial resolution of PGSE NV-NMR by optical means will reduce the signal to noise (SNR) ratio due to a decrease in the number of interrogated NV centers. For instance, increasing the spatial resolution by a factor of 2, will lead to a reduction of $\sim 2^3$ in the number of NV centers contributing to the detected signal (and the corresponding increase in averag-

ing time). We assume the following parameters for the lowest requirement for a PGSE NV-NMR experiment: six different gradient strength steps, averaged until a maximal SNR of 10 is reached (without an applied magnetic field gradient), with a duration of $t_{avr} = 3$ s for a single average. In Fig. S12 three different scenarios are displayed: 1) Current experimental conditions and sensitivity (η) using Overhauser dynamic nuclear hyperpolarization (ODNP); 2) current experimental conditions and η with thermal polarization of the nuclear spins (without ~ 200x enhancement by ODNP (*27*))); and 3) projected performance for a thermally polarized sample at an increased magnetic field of $B_0 = 1$ T and with an increased proton spin number sensitivity by a factor of ~ 180x. We assume improved fluorescence light collection ($\sim$10x, which translates to an sensitivity improvement $\sim$3x) (*60*), improved spin-state readout (we estimate a 10x improvement according to *Arunkumar et al.* (*32*)), and improved spectral linewidth (we estimate a 6x (3 to 0.5 Hz) improvement). It should be noted that the linewidth can be limited by susceptibility differences in heterogeneous (biological) samples (*59*), but it can also potentially be partly mitigated by careful susceptibility matching. Based on the projections in Fig. S12, it is evident that micron resolution can be achieved with ODNP in the current experiment. By incorporating the aforementioned technical advances and operating at 1 Tesla, comparable (micron) spatial resolution can be attained using a thermally polarized sample. This development paves the way for potential applications in biology and materials science. Especially the use of NV-imaging techniques, for which the photodiode and the necessary scanning of the laser location used in our experiments is replaced by a camera (*36*), might allow for a millimeter field of view while maintaining micron spatial resolution. We note, that at high spatial resolutions (few microns), statistical polarization may be dominant, making it increasingly difficult to record coherent NMR signals (*28, 62*). Here, the low number of sample spins within the detection volume, will cause random spin fluctuations (statistical polarisation or spin noise) to exceed thermal polarization by orders of magnitude. A more detailed explanation can be found

in Allert *et al.* (*28*).

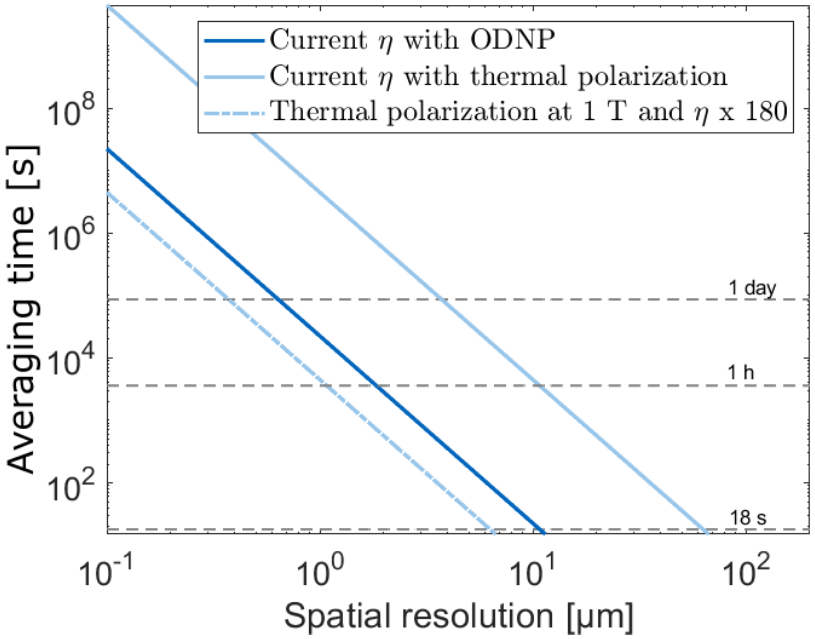

Figure S12: **Projected spatial resolution of PGSE NV-NMR.** The averaging time as a function of spatial resolution is shown for different sensitivities (η).

7 Laser and microwave induced sample heating

Since the ADC is temperature dependent (*43*), measuring the sample's temperature is important - in particular for NV-based experiments, where microwave or laser-induced heating can occur. However, measuring temperature in microfluidic channels is challenging due to small sample volumes. We utilized the NV center's ability to measure temperature, as the energy splitting of its two-level system is temperature-dependent (*70*). Under our experimental conditions, we are only able the probe a temperature change as a function of microwave and laser power. Moreover, the measured values give information on the temperature of the diamond - not of the sample (both are in thermal contact). We also note that the actual temperature may vary, depending on the experimental situation (*e.g.* position of the microwave loop, distance of the

measurement to the microwave loop etc.). Therefore, these values should be considered as a rough estimate.

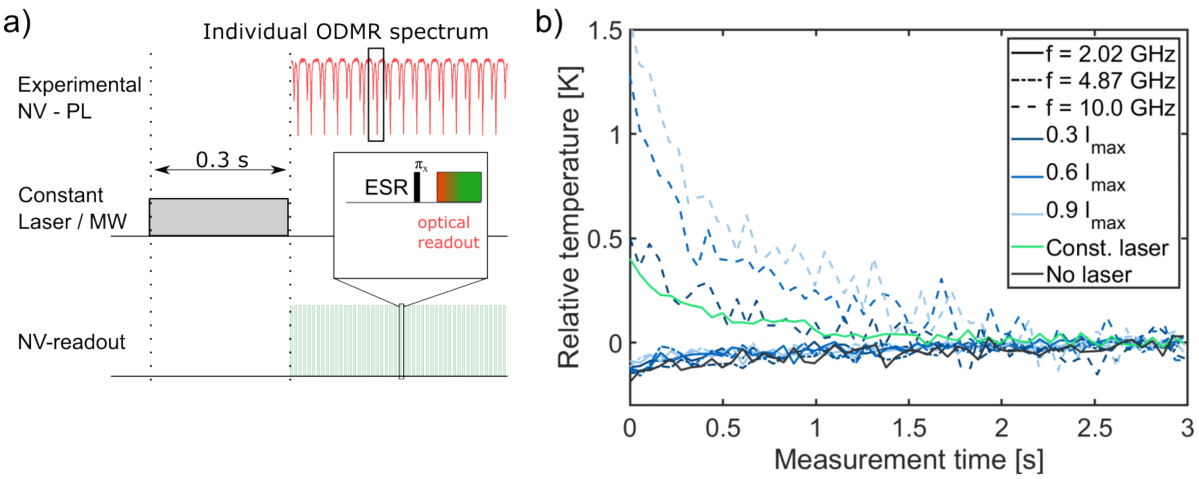

Figure S13: **Measurement of laser and MW induced sample heating. a)** The pulse sequence used for measuring the sample heating. A chain of ESR-experiments is used to detect the relative temperature of the diamond. Before the experiment a MW or laser pulse of length 0.3 s is used to heat the sample. Each individual ODMR spectrum is fitted, and the temperature during this spectrum is deduced from its midpoint. **b)** The relative temperature drift during the PGSE sequence as a function of time, as measured by the NV centers for various MW frequencies and powers tested.

In our experiment, we repeated the PGSE sequence, substituting the universally robust dynamical decoupling sequence with ESR sequences to determine the resonance frequency of the NV-ensemble. The MW frequency is swept between $f_0 \pm 5$ MHz, where f_0 is the resonance frequency of the NV centers and each pair of dips in NV-fluorescence corresponds to a single ODMR spectrum of the hyperfine splitting of the NV-centre's $|0\rangle \rightarrow |-1\rangle$ transition due to the ^{15}N-nucleus. Each individual spectrum is fit to a double Lorentzian model and f_0 is extracted. For each dataset, the mean of the last five datapoints is subtracted, to cancel out the effects of small temporal shifts in the magnetic field. Due to technical constraints we were only able to measure the relative change in temperature and not the absolute temperature.

To check microwave induced heating, we vary the amplitude I and frequency f of a 0.3

s long MW-pulse before our experiment, corresponding to the ODNP pulse in the NV-NMR-experiments. Since this microwave pulse has by far the longest duration and highest duty cycle, it is expected to produce the most significant heating effects. Results of these measurements can be seen in Fig. S13. For the MW-frequencies used in our experiments, $f \approx 2.02$ GHz and $f \approx 4.87$ GHz, we observe no significant heating effects. Only at higher frequencies, *e.g.* at $f \approx 10.0$ GHz, we see a significant heating effect of the NV-diamond.

To check for laser induced sample heating, the MW-pulse is replaced by a laser pulse. Here we observe small heating of ca. 0.5 K for a laser duty cycle of 100 % and no change when reducing the lasers duty cycle to 0%.

Since in our PGSE NV-NMR experiments much lower duty-cycles and/or amplitudes of both the laser and the MW are used, we exclude any excessive heating effects on our sample. Nevertheless, we anticipate minor heating that can influence the observed ADCs, as discussed in the main text.

Similar temperature calibration measurements as the ones done in this section could be performed by recording ^{1}H NMR spectra of ethylene glycol. However, only temperature drifts on a time scale longer than an NMR experiment could be recorded and fast temperature fluctuations induced by laser or microwave pulses would be invisible.

8 Individual ADC measurements

The ADC-tensors as calculated following Kingsley *et al.* (*37*).

$$ADC_{Loc1} = \begin{pmatrix} 2.24 & 0.14 & 0.07 \\ 0.14 & 1.93 & 0.29 \\ 0.07 & 0.29 & 2.16 \end{pmatrix}, \quad ADC_{Loc2} = \begin{pmatrix} 1.19 & 0.38 & 0.013 \\ 0.38 & 1.67 & 0.32 \\ 0.13 & 0.33 & 2.10 \end{pmatrix}, \tag{S10}$$

$$ADC_{Loc3} = \begin{pmatrix} 2.24 & 0.05 & 0.013 \\ 0.05 & 1.39 & 0.09 \\ 0.13 & 0.09 & 1.18 \end{pmatrix}$$

9 Chemical structures

We utilized two chemicals in the presented study: polyvinylpyrrolidone (PVP) and 4-Hydroxy-2,2,6,6-tetramethylpiperidin-1-oxyl (4-Hydroxy-TEMPO, TEMPOL). The chemical structures are shown in Fig. S14.

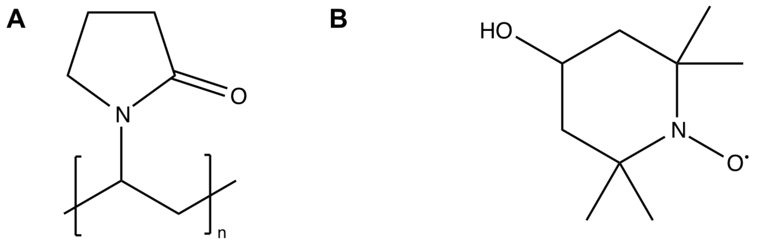

Figure S14: **Chemical structures of PVP and TEMPOL. A** Chemical structure of Polyvinylpyrrolidone (PVP). **B** Chemical structure of 4-Hydroxy-2,2,6,6-tetramethylpiperidin-1-oxyl (4-Hydroxy-TEMPO, TEMPOL)

.

10 Example data set of a measured NV-NMR PGSE experiment

An example data set of spin-echos are shown in Fig. S15, both in the time as well as in the frequency domain.

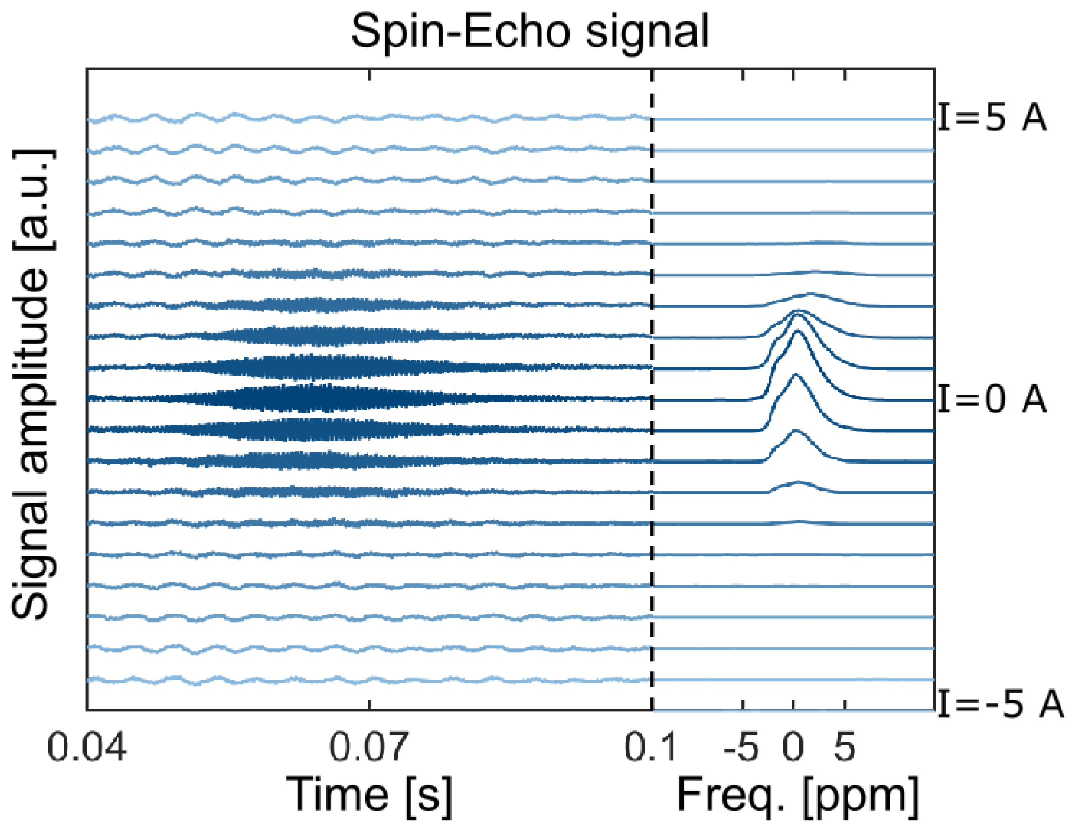

Figure S15: **Example measured dataset of an NV-NMR PGSE experiment.** A set of acquired spin-echos is displayed, both in the time-domain (left) as well as in the frequency-domain (right). This data set was measured using the $\hat{z}$-direction gradient coils at the location marked as "location 3" in the main manuscript. The current through the gradient coils was swept from -5 A (bottom) to 5 A (top). The increase and decrease of the signal amplitude is clearly visible.

REFERENCES AND NOTES

1. Reynold Spector, Nutrient transport systems in brain: 40 years of progress. *J. Neurochem.* **111**, 315–320 2009.
2. V. Gulani, A. G. Webb, I. D. Duncan, P. C. Lauterbur, Apparent diffusion tensor measurements in myelin-deficient rat spinal cords. *Magn. Res. Med.* **45**, 191–195 2001.
3. A. N. Landge, B. M. Jordan, X. Diego, P. Müller, Pattern formation mechanisms of self-organizing reaction-diffusion systems. *Dev. Biol.* **460**, 2–11 (2020).
4. R. M. Noyes, Models relating molecular reactivity and diffusion in liquids. *J. Am. Chem. Soc.* **78**, 5486–5490 1956.
5. T.-F. Yi, T.-T. Wei, Y. Li, Y.-B. He, Z.-B. Wang, Efforts on enhancing the li-ion diffusion coefficient and electronic conductivity of titanate-based anode materials for advanced li-ion batteries. *Energy Stor. Mater.* **26**, 165–197 (2020).
6. P. T. Callaghan, *Translational Dynamics and Magnetic Resonance* (Oxford Univ. Press, 2011).
7. J. J. Neil, Measurement of water motion (apparent diffusion) in biological systems. *Magn. Reson. A. Bridg.* **9**, 385–401 1997.
8. E. O. Stejskal, J. E. Tanner, Spin diffusion measurements: Spin echoes in the presence of a time-dependent field gradient. *J. Chem. Phys.* **42**, 288–292 (1965).
9. E. Fieremans, H.-H. Lee, Physical and numerical phantoms for the validation of brain microstructural MRI: A cookbook. *Neuroimage* **182**, 39–61 (2018).
10. G. A. Johnson, Y. Tian, D. G. Ashbrook, G. P. Cofer, J. J. Cook, J. C. Gee, A. Hall, K. Hornburg, Y. Qi, F.-C. Yeh, N. Wang, L. E. White, R. W. Williams, Merged magnetic resonance and light sheet microscopy of the whole mouse brain. *Proc. Natl. Acad. Sci.* **120**, e2218617120 (2023).
11. D. K. Jones, *Diffusion MRI* (Oxford Univ. Press, 2010).
12. D. L. Bihan, Looking into the functional architecture of the brain with diffusion MRI. *Nat. Rev. Neurosci.* **4**, 469–480, June 2003.
13. H. Lundell, C. Najac, M. Bulk, H. E. Kan, A. G. Webb, I. Ronen, Compartmental diffusion and microstructural properties of human brain gray and white matter studied with double diffusion encoding magnetic resonance spectroscopy of metabolites and water. *Neuroimage* **234**, 117981 (2021).
14. D. S. Novikov, E. Fieremans, S. N. Jespersen, V. G. Kiselev, Quantifying brain microstructure with diffusion MRI: Theory and parameter estimation. *NMR Biomed.* **32**, e3998 (2019).

15. P. T. Callaghan, C. D. Eccles, Y. Xia, NMR microscopy of dynamic displacements: k-space and q-space imaging. *J. Phys. E* **21**, 820–822 (1988).
16. S. Lasič, H. Lundell, D. Topgaard, T. B. Dyrby, Effects of imaging gradients in sequences with varying longitudinal storage time—Case of diffusion exchange imaging. *Mang. Res. Med.* **79**, 2228–2235 (2018).
17. G. Balasubramanian, I. Y. Chan, R. Kolesov, M. Al-Hmoud, J. Tisler, C. Shin, C. Kim, A. Wojcik, P. R. Hemmer, A. Krueger, T. Hanke, A. Leitenstorfer, R. Bratschitsch, F. Jelezko, J. Wrachtrup, Nanoscale imaging magnetometry with diamond spins under ambient conditions. *Nature* **455**, 648–651, October 2008.
18. J. R. Maze, P. L. Stanwix, J. S. Hodges, S. Hong, J. M. Taylor, P. Cappellaro, L. Jiang, M. V. Gurudev Dutt, E. Togan, A. S. Zibrov, A. Yacoby, R. L. Walsworth, M. D. Lukin, Nanoscale magnetic sensing with an individual electronic spin in diamond. *Nature* **455**, 644–647 (2008).
19. C. Müller, X. Kong, J.-M. Cai, K. Melentijević, A. Stacey, M. Markham, D. Twitchen, J. Isoya, S. Pezzagna, J. Meijer, J. F. Du, M. B. Plenio, B. Naydenov, L. P. McGuinness, F. Jelezko, Nuclear magnetic resonance spectroscopy with single spin sensitivity *Nat. Comun.* **5** 4703 (2014).
20. A. O. Sushkov, I. Lovchinsky, N. Chisholm, R. L. Walsworth, H. Park, M. D. Lukin, Magnetic resonance detection of individual proton spins using quantum reporters. *Phys. Rev. Lett.* **113**, 197601 (2014).
21. T. Staudacher, F. Shi, S. Pezzagna, J. Meijer, J. Du, C. A. Meriles, F. Reinhard, J. Wrachtrup, Nuclear magnetic resonance spectroscopy on a (5-nanometer)^3 sample volume. *Science* **339**, 561–563 (2013).
22. H. J. Mamin, M. Kim, M. H. Sherwood, C. T. Rettner, K. Ohno, D. D. Awschalom, D. Rugar, Nanoscale nuclear magnetic resonance with a nitrogen-vacancy spin sensor. *Science* **339**, 557–560 (2013).
23. K. S. Liu, A. Henning, M. W. Heindl, R. D. Allert, J. D. Bartl, I. D. Sharp, R. Rizzato, D. B. Bucher, Surface nmr using quantum sensors in diamond. *PNAS* **119**, e2111607119 (2021).
24. D. R. Glenn, D. B. Bucher, J. Lee, M. D. Lukin, H. Park, R. L. Walsworth, High-resolution magnetic resonance spectroscopy using a solid-state spin sensor. *Nature* **555**, 351–354 (2018).
25. N. Arunkumar, D. B. Bucher, M. J. Turner, P. T. Hon, D. Glenn, S. Lehmkuhl, M. D. Lukin, H. Park, M. S. Rosen, T. Theis, R. L. Walsworth, Micron-scale NV-NMR spectroscopy with signal amplification by reversible exchange. *PRX Quantum* **2**, 010305 (2021).
26. J. Smits, J. T. Damron, P. Kehayias, Andrew F. Mc Dowell, N. Mosavian, I. Fescenko, N. Ristoff, A. Laraoui, A. Jarmola, V. M. Acosta, Two-dimensional nuclear magnetic resonance spectroscopy with a microfluidic diamond quantum sensor. *Sci. Adv.* **5** eaaw7895 (2019).

27. D. B. Bucher, D. R. Glenn, H. Park, M. D. Lukin, R. L. Walsworth, Hyperpolarization-enhanced NMR spectroscopy with femtomole sensitivity using quantum defects in diamond. *Phys. Rev. X* **10**, 021053 (2020).
28. R. D. Allert, K. D. Briegel, D. B. Bucher, Advances in nano- and microscale NMR spectroscopy using diamond quantum sensors. *Chem. Commun.* **58**, 8165–8181 (2022).
29. R. D. Allert, F. Bruckmaier, N. R. Neuling, F. A. Freire-Moschovitis, K. S. Liu, C. Schrepel, P. Schätzle, P. Knittel, M. Hermans, D. B. Bucher, Microfluidic quantum sensing platform for lab-on-a-chip applications. *Lab Chip* **22**, 4831–4840 (2022).
30. F. Bruckmaier, K. D. Briegel, D. B. Bucher, Geometry dependence of micron-scale NMR signals on NV-diamond chips. *J. Magn. Reson. Open* **8-9**, 100023 (2021).
31. T. Wolf, P. Neumann, K. Nakamura, H. Sumiya, T. Ohshima, J. Isoya, J. Wrachtrup, Subpicotesla diamond magnetometry. *Phys. Rev. X* **5**, 041001 (2015).
32. N. Arunkumar, K. S. Olsson, J. T. Oon, C. Hart, D. B. Bucher, D. Glenn, M. D. Lukin, H. Park, D. Ham, R. L. Walsworth, Quantum logic enhanced sensing in solid-state spin ensembles. arXiv:2203.12501 (23 March 2022).
33. P. Amrein, F. Jia, M. Zaitsev, S. Littin, CoilGen: Open-source MR coil layout generator. *Magn. Reson. Med.* **88**, 1465–1479 (2022).
34. J.-P. Tetienne, L. Rondin, P. Spinicelli, M. Chipaux, T. Debuisschert, J.-F. Roch, V. Jacques, Magnetic-field-dependent photodynamics of single NV defects in diamond: An application to qualitative all-optical magnetic imaging. *N. J. Phys.* **14**, 103033 (2012).
35. P. Amrein, F. Bruckmaier, F. Jia, D. B. Bucher, M. Zaitsev, S. Littin, Optimal bi-planar gradient coil configurations for diamond nitrogen-vacancy based diffusion-weighted NMR experiments. *Magn Reson Mater Phy.* https://doi.org/10.1007/s10334-023-01111-0 (2023)
36. E. V. Levine, M. J. Turner, P. Kehayias, C. A. Hart, N. Langellier, R. Trubko, D. R. Glenn, R. R. Fu, L. Ronald, Walsworth. Principles and techniques of the quantum diamond microscope. *Nanophotonics* **8**, 1945–1973 (2019).
37. P. B. Kingsley, Introduction to diffusion tensor imaging mathematics: Part III. Tensor calculation, noise, simulations, and optimization. *Magn. Reson. A* **28A**, 155–179 (2006).
38. P. T. Callaghan, Y. Xia, Velocity and diffusion imaging in dynamic NMR microscopy. *J. Magn. Reson.* **91**, 326–352 (1991).

39. N. H. Williamson, M. E. Komlosh, D. Benjamini, P. J. Basser, Limits to flow detection in phase contrast MRI. *J. Magn. Reson. Open* **2-3**, 100004 (2020).
40. R. Mills, Self-diffusion in normal and heavy water in the range 1-45.deg. *J. Phys. Chem.* **77**, 685–688 (1973).
41. K. R. Harris, L. A. Woolf, Pressure and temperature dependence of the self diffusion coefficient of water and oxygen-18 water. *J. Chem. Soc. Faraday Trans. 1* **76**, 377–385 (1980).
42. P. S. Tofts, D. Lloyd, C. A. Clark, G. J. Barker, G. J. M. Parker, P. McConville, C. Baldock, J. M. Pope, Test liquids for quantitative MRI measurements of self-diffusion coefficient in vivo. *Magn. Reson. Med.* **43**, 368–374 (2000).
43. F. Wagner, F. B. Laun, T. A. Kuder, A. Mlynarska, F. Maier, J. Faust, K. Demberg, L. Lindemann, B. Rivkin, A. M. Nagel, M. E. Ladd, K. Maier-Hein, S. Bickelhaupt, M. Bach, Temperature and concentration calibration of aqueous polyvinylpyrrolidone (PVP) solutions for isotropic diffusion MRI phantoms. *PLOS ONE* **12**, e0179276 (2017).
44. J. Eills, W. Hale, M. Utz, Synergies between hyperpolarized NMR and microfluidics: A review. *Prog. Nucl. MAgn. Reson. Scpectros.* **128**, 44–69 (2022).
45. R. Kimmich, W. Unrath, G. Schnur, E. Rommel, NMR measurement of small self-diffusion coefficients in the fringe field of superconducting magnets. *J. Magn. Reson.* **91**, 136–140 (1991).
46. P. T. Callaghan, M. E. Komlosh, M. Nyden, High magnetic field gradient PGSE NMR in the presence of a large polarizing field. *J. Magn. Reson.* **133**, 177–182 (1998).
47. H. Zhang, K. Arai, C. Belthangady, J.-C. Jaskula, R. L. Walsworth, Selective addressing of solid-state spins at the nanoscale via magnetic resonance frequency encoding *npj Quant. Inf.* **3**, 31 (2017).
48. K. Arai, C. Belthangady, H. Zhang, N. Bar-Gill, S. J. DeVience, P. Cappellaro, A. Yacoby, R. L. Walsworth, Fourier magnetic imaging with nanoscale resolution and compressed sensing speed-up using electronic spins in diamond. *Nat. Nanotechnol.* **10**, 859–864 (2015).
49. F. Szczepankiewicz, C.-F. Westin, M. Nilsson, Maxwell-compensated design of asymmetric gradient waveforms for tensor-valued diffusion encoding. *Magn. Reson. Med.* **82**, 1424–1437 (2019).
50. H. H. Heenen, C. Scheurer, K. Reuter, Implications of occupational disorder on ion mobility in li$-4ti_5o_{12}$ battery materials. *Nano Lett.* **17**, 3884–3888 (2017).
51. P. Benedek, O. K. Forslund, E. Nocerino, N. Yazdani, N. Matsubara, Y. Sassa, F. Jurànyi, M. Medarde, M. Telling, M. Månsson, V. Wood, Quantifying diffusion through interfaces of lithium-ion battery active materials. *ACS Appl. Mater. Interf.* **12**, 16243–16249 (2020).

52. C. M. W. Tax, F. Szczepankiewicz, M. Nilsson, D. K. Jones, The dot-compartment revealed? diffusion MRI with ultra-strong gradients and spherical tensor encoding in the living human brain. *Neuroimage* **210**, 116534 (2020).
53. I. O. Jelescu, L. Ciobanu, F. Geffroy, P. Marquet, D. L. Bihan, Effects of hypotonic stress and ouabain on the apparent diffusion coefficient of water at cellular and tissue levels in *Aplysia*. *NMR Biomed.* **27**, 280–290 (2014).
54. D. Wu, J. Xu, M. T. McMahon, P. C. M. van Zijl, S. Mori, F. J. Northington, J. Zhang, In vivo high-resolution diffusion tensor imaging of the mouse brain. *Neuroimage* **83**, 18–26 (2013).
55. P. Schätzle, P. Reinke, D. Herrling, A. Götze, L. Lindner, J. Jeske, L. Kirste, P. Knittel, A chemical vapor deposition diamond reactor for controlled thin-film growth with sharp layer interfaces. *Phys. Status Solidi* **220**, 2200351 (2022).
56. D. B. Bucher, D. P. L. Aude Craik, M. P. Backlund, M. J. Turner, O. B. Dor, D. R. Glenn, R. L. Walsworth, Quantum diamond spectrometer for nanoscale NMR and ESR spectroscopy. *Nat. Protoc.* **14**, 2707–2747 (2019).
57. R. A. Lemdiasov, R. Ludwig, A stream function method for gradient coil design. *Concepts Magn. Reson. Part B Magn. Reson. Eng.* **26B**, 67–80 (2005).
58. G. T. Genov, D. Schraft, N. V. Vitanov, T. Halfmann, Arbitrarily accurate pulse sequences for robust dynamical decoupling. *Phys. Rev. Lett.* **118**, 133202 (2017).
59. T. A. A. Cartlidge, T. B. R. Robertson, M. Utz, G. Pileio, Theory and simulation framework for the relaxation of nuclear spin order in porous media. *J. Phys. Chem. B* **126**, 6536–6546 (2022).
60. N. Neuling, R. D. Allert, D. B. Bucher, Prospects of single-cell nuclear magnetic resonance spectroscopy with quantum sensors, *Current Opinion in Biotechnology* **83**, 102975 (2023)
61. C. L. Degen, F. Reinhard, P. Cappellaro, Quantum sensing. *Rev. Mod. Phys.* **89**, 035002 (2017).
62. R. Rizzato, F. Bruckmaier, K. S. Liu, S. J. Glaser, D. B. Bucher, Polarization transfer from optically-pumped NV center ensembles to multinuclear spin baths. *Phys. Rev. Applied* **17**, 024067 (2022).
63. J. F. Barry, J. M. Schloss, E. Bauch, M. J. Turner, C. A. Hart, L. M. Pham, R. L. Walsworth, Sensitivity optimization for NV-diamond magnetometry. *Rev. Mod. Phys.* **92**, 015004 (2020).
64. M. W. Doherty, N. B. Manson, P. Delaney, F. Jelezko, J. Wrachtrup, L. C. L. Hollenberg, The nitrogen-vacancy colour centre in diamond. *Phys. Rep.* **528**, 1–45 (2013).
65. H. Y. Carr, E. M. Purcell, Effects of diffusion on free precession in nuclear magnetic resonance experiments. *Phys. Rev.* **94**, 630–638 (1954).

66. F. Reinhard, *Nanoscale Sensing and Quantum Coherence* (IOS Press, 2019).
67. P. P. Mitra, P. N. Sen, L. M. Schwartz, Short-time behavior of the diffusion coefficient as a geometrical probe of porous media. *Phys. Rev. B* **47**, 8565–8574 (1993).
68. J. E. Tanner, E. O. Stejskal, Restricted self-diffusion of protons in colloidal systems by the pulsed-gradient, spin-echo method. *J. Chem. Phys.* **49**, 1768–1777 (1968).
69. A. Kusumi, Y. Sako, M. Yamamoto, Confined lateral diffusion of membrane receptors as studied by single particle tracking (nanovid microscopy). effects of calcium-induced differentiation in cultured epithelial cells. *Biophys. J.* **65**, 2021–2040 (1993).
70. M. Fujiwara, Y. Shikano, Diamond quantum thermometry: From foundations to applications. *Nanotechnology* **32**, 482002 (2021).
